# A Review Into the Insights of the Role of Endothelial Progenitor Cells on Bone Biology

**DOI:** 10.3389/fcell.2022.878697

**Published:** 2022-05-24

**Authors:** Henglei Shi, Zhenchen Zhao, Weidong Jiang, Peiqi Zhu, Nuo Zhou, Xuanping Huang

**Affiliations:** ^1^ Department of Oral and Maxillofacial Surgery, Hospital of Stomatology, Guangxi Medical University, Nanning, China; ^2^ Guangxi Key Laboratory of Oral and Maxillofacial Rehabilitation and Disease Treatment, Guangxi Clinical Research Center for Craniofacia Reconstruction, Guangxi Key Laboratory of Oral and Maxillofacial Surg Deformity, Nanning, China

**Keywords:** endothelial progenitor cells, bone biology, bone regeneration, bone vascularization, bone immunity, interaction

## Abstract

In addition to its important transport functions, the skeletal system is involved in complex biological activities for the regulation of blood vessels. Endothelial progenitor cells (EPCs), as stem cells of endothelial cells (ECs), possess an effective proliferative capacity and a powerful angiogenic capacity prior to their differentiation. They demonstrate synergistic effects to promote bone regeneration and vascularization more effectively by co-culturing with multiple cells. EPCs demonstrate a significant therapeutic potential for the treatment of various bone diseases by secreting a combination of growth factors, regulating cellular functions, and promoting bone regeneration. In this review, we retrospect the definition and properties of EPCs, their interaction with mesenchymal stem cells, ECs, smooth muscle cells, and immune cells in bone regeneration, vascularization, and immunity, summarizing their mechanism of action and contribution to bone biology. Additionally, we generalized their role and potential mechanisms in the treatment of various bone diseases, possibly indicating their clinical application.

## Introduction

The functional state of bone, including the physiological state of bone formation and regeneration and the pathological state of bone resorption and remodeling, significantly impacts human health. In the case of extensive bone disorders caused by major diseases and traumatic injuries, it is difficult for bones to repair themselves (Li et al., 2015). With further advancements in tissue engineering and stem cell research, we have observed efficient solutions to these problems. Previous osteobiological studies mostly focused on osteogenesis and related regulation of mesenchymal stem cells (MSCs); however, with the advancement in vascularization studies, blood vessels were observed to be indispensable in bone activity (Maes et al., 2010; Percival and Richtsmeier, 2013). Consequently, endothelial progenitor cells (EPCs), which possess a strong angiogenic ability, have received sufficient attention (George et al., 2011). Hence, based on the current studies, our review focuses on the interaction and benefits of EPCs in bone regeneration, vascularization, and immunity and discusses the lack of research on osteoclastogenesis and bone hemodynamics.

For bone regeneration, captivated by most researchers, early treatment strategies focused on the construction of different scaffolds and the transplantation of MSCs (Quarto et al., 2001). However, in the absence of a functional vascular network, with implantation of scaffolds or MSCs alone, rapid healing of bone was difficult to achieve, since MSCs demonstrated an insufficient number of integrated cells and death at an early stage (Pang et al., 2013; Lin et al., 2014; Kim et al., 2021). Successful bone regeneration and vascularization have proven to be inextricably linked (Grosso et al., 2017). Furthermore, EPCs, as precursors of endothelial cells (ECs), possess strong proliferative and angiogenic abilities. Different strategies have been applied to bone tissue engineering for vascularization, including single/multiple cell transplantation, growth factors, prevascularization of grafts, and co-culturing (Zhuang et al., 2021). The co-transplantation of MSCs and EPCs has an effective synergistic effect on vascularization and bone regeneration. EPCs and MSCs mutually co-regulate each other by secreting multiple growth factors to promote early angiogenesis and bone reconstruction (Bouland et al., 2021).

The contribution of EPCs to bone vascularization is an important topic to be considered. In addition to differentiating into ECs, EPCs also play a direct regulatory role in the development of ECs. Additionally, the subtypes of ECs, H and L subtypes, form subtypes of bone microvessels, which have a significant impact on osteogenesis and osteoclastogenesis (Kusumbe et al., 2014). In addition, capillaries invade the initial ossification site during the early stages of intramembranous and endochondral ossification, providing essential factors like oxygen and modifying osteogenesis (Percival and Richtsmeier, 2013). Although a majority of blood vessels in the skeleton are capillaries, several intact vascular structures are also present (Prisby, 2020). Since smooth muscle cells (SMCs) are responsible for stabilizing blood vessels, whether there is an interaction between SMCs and EPCs should be explored.

**FIGURE 1:**
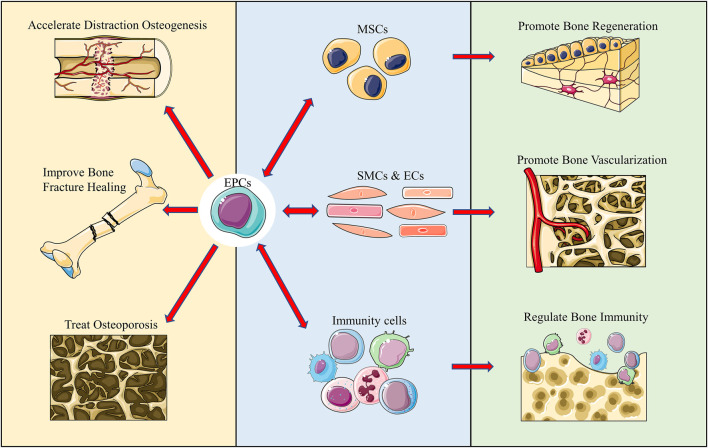
Graphical abstract. We reviewed and summarized the complex interactions of EPCs with multiple cells in bone regeneration, vascularization, and immunity. Additionally, we summarized the application of EPCs in various disease models and believe that it offers certain directions for future studies.

Immune regulation also plays an important role in the biological activity of the bone. Immune cells, including neutrophils, monocytes, and macrophages, are enriched in the skeletal system. Neutrophils are recruited to the wounded area at an early stage, releasing inflammatory factors and proteolytic enzymes to promote tissue reconstruction (Franz et al., 2011). In the later stage, M2 macrophages secrete tissue repair factors, which recruit MSCs and promote angiogenesis (Schlundt et al., 2018). In this review, we, first, describe the definition and classification of EPCs and second, focus on the interactions and EPCs with different cells and their effects, which ultimately affect bone biology. We believe that elucidation of the molecular mechanisms involved in bone metabolism by EPCs will not only elaborate our understanding of bone-related diseases but also provide potential research directions and therapeutic approaches for their treatment (Figure 1; Table 1).

**TABLE 1:** Outcomes and mechanisms of interaction between EPCs and other cells in bone biology.

Cell type	*In vivo*/*in vitro*	Outcomes		Mechanisms	References
MSCs	*In vitro*		MSCs preservation of stemness	The upregulation of stem regulators, OCT4, SOX2, Nanog, and Klf4	Wen et al. (2016)
	*In vivo*		MSCs reservation of regenerative capacity in the early stage	EPCs secretion of PDGF-BB	Lin et al. (2014)
	*In vitro*		MSCs differentiation toward osteogenesis	EPCs secretion of BMP-2 and the activation of the MAPK signaling pathway	Murphy et al. (2016), Xu et al. (2020)
	*In vitro*		EPCs enhancement of migration, invasion, and vessel forming	MSCs secretion of PDGF-BB、IGF-1、SDF-1	Kamprom et al. (2016a), Kamprom et al. (2016b)
	*In vivo* and *in vitro*		EPCs mobilization	MSCs secretion of CXCR2 ligands, activating the Src-PKL/Vav2-Rac1	Li et al. (2011), Li et al. (2018)
	*In vivo* and *in vitro*		EPCs proliferation	MSCs secretion of SDF-1, activating CXCR4/SDF-1 pathway	Hattori et al. (2001), Heissig et al. (2002), Walter et al. (2005), Keshavarz et al. (2019)
	*In vivo*		EPCs migration and function	MSCs secretion of a low dose of SDF-1α	Premer et al. (2019)
	*In vivo*		EPCs proliferation, migration, and angiogenic differentiation	MSC-EXOs are abundant in miR-21, increasing the expression of VEGF-A and HIF-1α	Zhang et al. (2021)
	*In vivo* and *in vitro*		EPCs rejuvenation of aging and angiogenic improvement	MSC-EVs contained miR-126, inhibiting Spred-1	Liyong Wang et al. (2020)
	*In vivo* and *in vitro*		EPCs angiogenesis	EPCs and MSCs adhesion by the recognition of endoglin and integrin on the surfaces	Rossi et al. (2017)
SMCs	*In vivo* and *in vitro*		EPCs improved survival and more stable vascular network	SMPCs secretion of Ang-1, activating the receptor Tie-2	Foubert et al. (2008)
	*In vivo* and *in vitro*		The exerting release of angiogenic factors and more anti-apoptotic and anti-fibrotic properties	SMC-EPC bi-level cell sheet, by the direct junction and potential cytokine communication	Shudo et al. (2013), Shudo et al. (2017), Kawamura et al. (2017)
	*In vivo* and *in vitro*		Alleviation of SMCs transition to a synthetic phenotype	EPCs-EXOs transportation of functional ACE2 and lessening the activation of the NF-κB pathway	Yang et al. (2019), Jinju Wang et al. (2020)
ECs	*In vivo* and *in vitro*		Vascular remodeling and ECs proliferation	EPCs secretion of Ang, SDF-1, PDGF-BB, VEGF, and MMP	Di Santo et al. (2009), Maki et al. (2018)
	*In vitro*		ECs proliferation, migration, and capillary sprouting	EPC-CM induction of the activation of PI3K/AKT and MEK/ERK pathways	Di Santo et al. (2014)
	*In vitro*		ECs cytoprotective properties	EPCs secretion of paracrine factors in intracellular antioxidant defense and pro-survival signals	Yang et al. (2010)
	*In vivo*		ECs inhibition of apoptosis	EPCs decreased the expression of PUMA and augmented the expression of Bcl-2	Liang et al. (2015)
	*In vivo* and *in vitro*		ECs enhanced phenotypic changes and angiogenesis	EPC-EXOs transportation of miR-1246 and miR-1290, targeting ELF5 and SP1	Yulang Huang et al. (2021)
	*In vivo* and *in vitro*		ECs anti-apoptotic effect and the stimulation of the organization	EPC-MVs transportation of adhesion molecules like ICAM-1, α4 integrin, CD44, and CD29, activating PI3K/Akt signaling pathway and eNOS	Deregibus et al. (2007)
	*In vivo* and *in vitro*		ECs proliferation, angiogenesis, and antiapoptosis	EPCs and EPC-EXOs containing IL-10, *via* the miR-375/PDK-1 signaling axis and NF-κB signaling	Yue et al. (2020)
	*In vivo*		ECs improved angiogenesis	MiR-21-5p affluence in EPC-EXOs, inhibiting THBS1	Hu et al. (2019)
	*In vivo* and *in vitro*		ECs antiapoptosis under hypoxia environment	EPC-MVs deregulation of inflammatory and proapoptotic caspases	Deregibus et al. (2007)
MMs	*In vivo* and *in vitro*		M1 MMs activation reduction without the change of M2 MMs	EPC-CM alleviation of the expression of IL-1β and IL-6	Wang et al. (2018)
	*In vivo* and *in vitro*		MMs migration and osteoclast differentiation	EPCs secretion of TGF-β1, binding to β integrins on the MMs surface, upregulating Talin-1 expression	Cui et al. (2018)
	*In vivo* and *in vitro*		MMs enhanced infiltration	EPCs high expression of E-selectin, increasing adhesion to MMs	Chen et al. (2018)
T cells	*In vitro*		T cells proliferation suppression	EPCs down-modulation of CD4^+^ and CD8^+^ T cells activation, *via* the TNF-α/TNFR2 pathway	Naserian et al. (2020)
Tang	*In vivo* and *in vitro*		Constitution of EPC colonies and EPCs differentiation	Tang secretion of high levels of VEGF, IL-8, IL-17, MMP, and G-CSF	Hur et al. (2007)
NK cells	*In vivo* and *in vitro*		EPCs lysis augmentation	NK cells production of granzyme and the recognition of CX3CL1 on EPCs	Sehgal et al. (2020)
Neutrophils	*In vivo* and *in vitro*		EPCs mobilization	Neutrophils generation of VEGF	Ohki et al. (2005)
	*In vitro*		EPCs migration	Leucocytes secretion of elastase, targeting VEGF-A to form VEGFf	Kurtagic et al. (2015)
	*In vivo* and *in vitro*		EPCs activation and increased angiogeneses	EPCs and neutrophils communicate *via* the recognition of PSGL-1 and L-selectin	Hubert et al. (2014)
	*In vivo* and *in vitro*		EPCs impairment	EPCs adhesion to leucocytes *via* CD18 and CD54, leucocytes secretion of ROS	Henrich et al. (2011)
	*In vivo* and *in vitro*		Leucocytes activation and transmigration	Leucocytes’ adhesion to EPCs *via* endoglin and integrin-α5β1	Rossi et al. (2013)
	*In vivo* and *in vitro*		Neutrophils diminished infiltration	EPC-MVs abundant in miRs attenuating inflammatory factors	Cantaluppi et al. (2012)

## Definition and Classification of EPCs

EPCs, circulating cells considered to be primarily located in the bone marrow albeit in minor quantities in the peripheral blood, were first isolated from human peripheral blood by magnetic bead sorting and suggested to augment collateral vessel growth (Asahara et al., 1997). EPCs are mobilized into the circulation and directed to tissue sites in response to multiple cytokines and signals under trauma, ischemia, and tissue remodeling conditions (George et al., 2011). Owing to their unique functions in a vascular generation, their subsets and potential functions and mechanisms have been studied. EPCs are defined as precursor cells enabled to differentiate into ECs and SMCs (Miyata et al., 2005; Sai et al., 2014). EPCs have two typical features in the biological process: Clonal expansion and stemness. However, per the experimental observations, EPCs are mainly considered to be mononuclear cells that attach to matrix molecules, dually positive for acetylated low-density lipoprotein, and *Ulex europaeus* agglutinin lectin in cell-culture studies (Huang Z. et al., 2021).

Following long-term studies and debates, the researchers grouped the different subsets of EPCs and organized them into two major categories based on their hematopoietic or endothelial lineage (Medina et al., 2017). Myeloid angiogenic cells (MACs), of hematopoietic lineage, do not differentiate into ECs albeit derive paracrine factors as stimulants to promote angiogenesis. MACs are considered to be generated from peripheral blood monocytes under endothelial cell culture conditions and have weak proliferation capacity, which causes difficulty in the passage (Chambers et al., 2018). Endothelial colony-forming cells (ECFCs), of endothelial lineage, differentiate into ECs, exhibit pronounced proliferative capacity with the potential to develop vascular networks, and promote the recovery of wounded endothelium and angiogenesis (Tasev et al., 2016). The most commonly used markers of MACs are used in combinations of CD45+/CD14+/CD31+/vascular endothelial growth factor receptor 2 (VEGFR2) +/CD146-/CD34-, as CD31+/CD146+/VEGFR2+/CD45-/CD14- for ECFCs (Medina et al., 2017). Secondary to the functional similarity of MACs and ECFCs, most researchers could not accurately distinguish between the two yet; therefore, this will be discussed in this review in uniformity with EPCs.

## Interaction Between EPCs and Other Cells in Bone Biology

### Interaction of EPCs in Bone Regeneration

MSCs play an undoubtedly important role in bone regeneration. They are a type of pluripotent stem cells that exhibit great potential for differentiation into various lineages, including osteoblasts, chondrocytes, myocytes, and adipocytes. Almost all tissues in the human body contain MSCs, especially those of bone marrow, fat, dental pulp, umbilical cord, and placenta (Bouland et al., 2021). MSCs actively translocate to the site of tissue damage and participate in immune regulation and tissue damage repair. These properties provide MSCs with great potential for application in the domain of histological engineering and regenerative medicine, making them clinically valuable stem cells for cell therapy. Numerous patients have been enrolled in various clinical trials, and no serious adverse events have been reported so far (Watson et al., 2014). However, ECs and related cell lines are rarely used as therapeutic agents in phase I studies owing to hesitation by investigators in using them, complexity, and security risks. Meanwhile, in the process of bone formation, the coupling of angiogenesis and osteogenesis has been taken into consideration. Secondary to the stemness of EPCs and MSCs, the effect of co-culture and the interaction between MSCs and EPCs have been investigated. The synergistic effect, improved bone formation, and higher and earlier neovascularization were observed in the co-culture of bone marrow-MSCs/EPCs.

In the co-cultivation system of EPCs and MSCs, EPCs not only influence the function of MSCs but also secrete factors to impact their function. By EPCs/MSCs indirect transwell co-culture system, co-cultured MSCs preserve stemness without any morphological changes. In addition to the enhanced proliferation, expressions of the core regulators of stemness, OCT4, SOX2, Nanog, and Klf4, were upregulated in co-cultured MSCs (Wen et al., 2016). EPCs nourished MSCs prior to the neovascularization and hemoperfusion, which prevented apoptosis of MSCs in the early stage (Lin et al., 2014). With the EPC secretion of platelet-derived growth factor-BB (PDGF-BB), PDGFR-β+ MSCs reserve vigorous regenerative capacity, whereas PDGFR-β- MSCs lose stem cell properties (Lin et al., 2014). Moreover, EPCs secrete bone morphogenetic protein-2 (BMP-2) as a paracrine signaling molecule to contribute to osteogenic differentiation whereas MSCs do not secrete BMP-2 alone (Murphy et al., 2016). The soluble factors secreted by ECs selectively stimulate MSC differentiation activity with the upregulation of *alkaline phosphatase*, *BMP-2*, *osteonectin*, and *osteopontin* genes (Saleh et al., 2011). Additionally, Xu et al. determined the action of the EPC signaling pathway. Through microarray analysis and Kyoto Encyclopedia of Genes and Genomes enrichment analysis of co-cultured versus individually cultured MSCs and further validation, they determined that EPCs assist osteogenic differentiation of MSCs mainly by upregulating TAB1 to promote p38 phosphorylation. The mitogen-activated protein kinase (MAPK) signaling pathway was majorly affected by co-cultivation with EPCs, and extracellular-signal-regulated kinase (ERK)1/2, and c-Jun N-terminal kinase (JNK) pathways were activated by the upregulation of TAB1, which promoted p38 phosphorylation by direct combination. They further observed increased phosphorylation in the downstream MAPK signaling pathway in contrast with that in ERK1/2 and JNK pathways, as confirmed by the utilization of their respective inhibitors (Xu et al., 2020). Transplanted EPCs release chemokines such as VEGF, recruit host EPCs and stimulate angiogenesis at the bone defect with EPCs-MSCs loaded on β-TCP *in vivo* (Seebach et al., 2010). Joo et al. reported that VEGFR2 phosphorylation and induction of angiogenic buds in EPCs stimulated by low doses of VEGF-A may contribute to their stronger angiogenic potential (Joo et al., 2015). Additionally, extracellular vesicles (EVs) as a novel mediator of intercellular interaction pathways have received considerable attention. According to their diameters, EVs are commonly divided into exosomes (EXOs), microvesicles (MVs), and apoptotic bodies (Wu et al., 2021). EVs carry multidimensional biomolecules, cross biological barriers, mediate information exchange among cells, and avoid phagocytosis by macrophages, which hold promising potential for tissue regeneration and repair (Zhang K.-L. et al., 2019). The proliferation and migration of osteoblast precursor cells, MC3T3-E1, are promoted by EPC-MVs while the simultaneous reduction of apoptosis. A study regarded the microRNA-126 (miR-126) enrichment in EPC-MVs as the key to amplifying beneficial effects, which simultaneously enhanced the expression of Bcl-2 and p-Erk1/2, indicating the potential activation of the Erk1/2-Bcl-2 signal (Chen et al., 2019).

MSCs also modulate EPCs. The presence of MSCs supports the differentiation of EPCs into a more mature endothelial cell phenotype at an early stage. Following MSCs implantation, Seebach et al. also observed more host-cell attraction and proangiogenic activity of EPCs (Seebach et al., 2014). Through mass spectrometry and filtering, Kamprom et al. predicted a unique combination of factors enhancing the effects of EPC derived from placental-derived MSCs, including 12 proteins (Kamprom et al., 2016b). They further recognized the varied functions and action modes of rich sources of MSCs. Placenta-derived MSCs exhibited the highest migration progress with the secretion of PDGF-BB. Insulin-like growth factor-1 and stromal cell-derived factor (SDF)-1 were detected when bone marrow-derived MSCs achieved the maximal enhancement of invasion and vessel formation (Kamprom et al., 2016a). Under inflammatory microenvironments, MSCs significantly enhance the release of C-X-C chemokine receptor (CXCR) 2 ligands, which perform the critical function of the mobilization of EPCs (Li et al., 2011; Li et al., 2018). Although CXCR2-mediated migration of EPCs may be mediated by multiple signaling pathways, Src has been reported to be a leading downstream effector of CXCR2 through respective inhibitors for *in vitro* migration assays. Additional work revealed Rac1 to be the downstream effector of CXCR2-Src, which was modulated by paxillin kinase linker and Vav2 (Li et al., 2018). Furthermore, Keshavarz et al. revealed secretion of the SDF-1 by MSCs on the proliferation latency of bone marrow-derived EPCs (Keshavarz et al., 2019). SDF-1 upregulated its receptor, CXCR4, and this interaction activates bone MSCs, causing the generation of matrix metalloproteinases-9 (MMP-9) (Hattori et al., 2001; Walter et al., 2005). Following the release of soluble kit-ligand into the extracellular matrix, a recipient for c-kit expressed on the membrane of EPCs causes the combination and migration of c-kit + EPCs from the cell into circulation (Heissig et al., 2002). Additionally, EPCs stimulate homing of ECs and mesenchymal cells to accelerate osteogenesis by secreting SDF-1 and activating the CXCR4/SDF-1 pathway (Tamari et al., 2020). In contrast, Premer et al. detected that a low level of SDF-1α secreted by MSCs decreased the elevated levels of tumor necrosis factor-α (TNF-α) and enhanced EPC function in a dose-dependent manner whereas a high level of SDF-1α hindered the benefits (Premer et al., 2019). MSC-EXOs enhance the features of EPCs but do not notably influence the ossification of MSCs (Zhang et al., 2021). The high-throughput sequencing suggested miR-21 abundance in MSC-EXOs, which mimics the increased VEGF-A and hypoxia-inducible factor 1-alpha (HIF-1α) expression but reduced *NOTCH1* and *delta-like 4* (*DLL4*) expression, indicating the potential regulation of the *NOTCH1*/*DLL4* pathway (Zhang et al., 2021). Interestingly, in addition to EPCs, MSCs also generate EVs carrying miR-126 (Wang L. et al., 2020). MiR-126 has been confirmed as an influential participant in the maintenance of EC function and the promotion of vascularization (Wang et al., 2008). MiR-126-containing MSC-EVs are consumed by EPCs and inhibit the expression of Spred-1, a key target gene of miR-126 and an endogenous inhibitor of VEGF signaling in EPCs. Therefore, even EVs produced by senescent MSCs rejuvenate old EPCs *in vitro* and improve angiogenesis *in vivo* (Wang L. et al., 2020).

**FIGURE 2:**
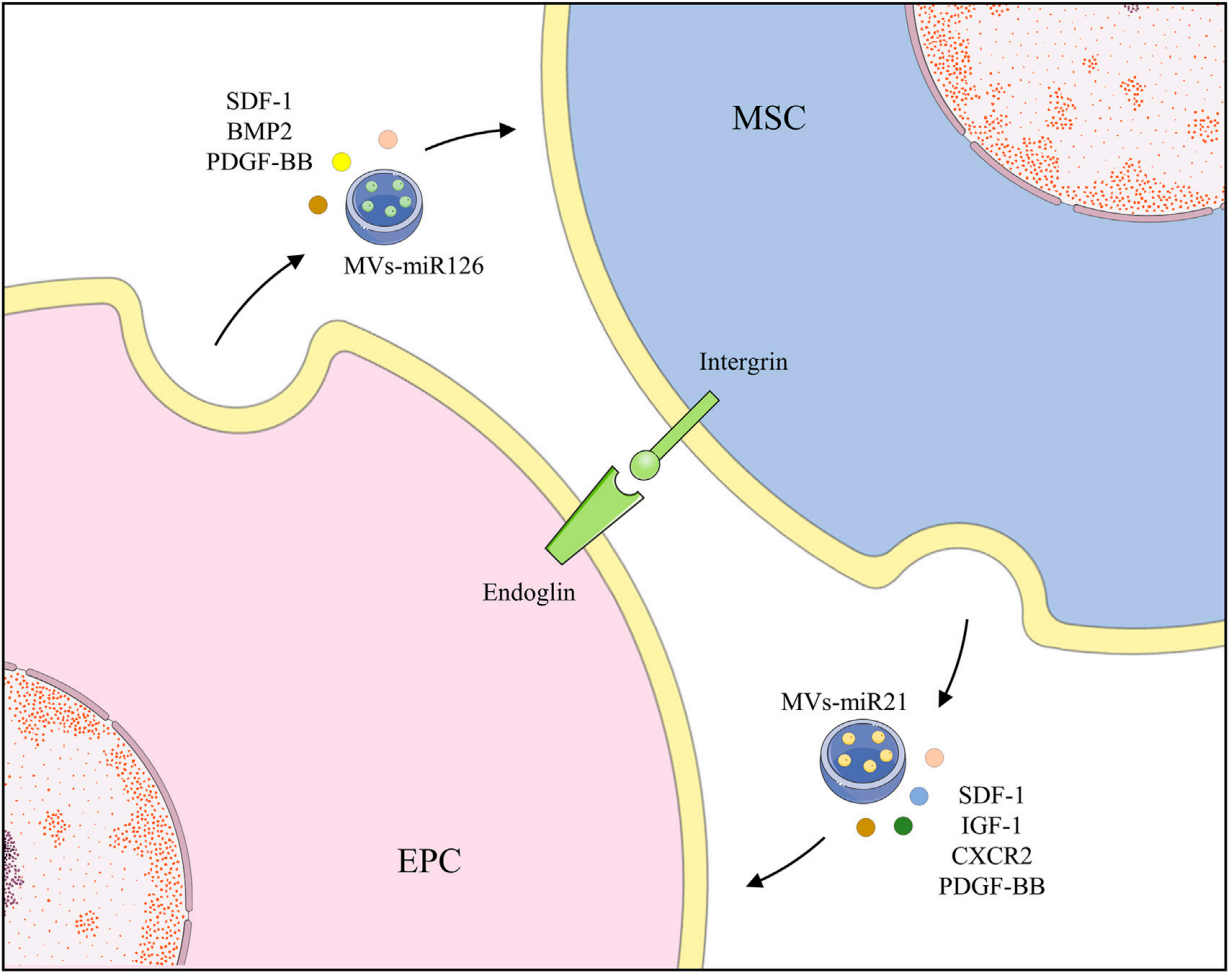
EPCs and MSCs recognize and bind to each other through endoglin and secrete various growth factors and EVs to promote functions of each other.

Most of the aforementioned studies focused on how paracrine factors and EVs communicate, however, regulation of their direct contact action is still unknown. This is because cell separation becomes difficult when they are connected. However, cells are likely to be in direct contact in the microenvironment of bone marrow. Thus, it is essential to explore how they are connected and moderated. Co-transplantation of MSCs and EPCs accelerated recovery in an ischemia model *via* an endoglin-dependent manner, in which silencing EPCs remarkably inhibited adhesion to MSCs (Rossi et al., 2017). Endoglin, also termed CD105, is a type I transmembrane glycoprotein notably expressed in ECs and EPCs and containing the arginine-glycine-aspartic acid (RGD) region (Rossi et al., 2019). It is called a critical co-receptor of the transforming growth factor β (TGF-β) family and a key mediating factor in angiogenesis and cell adhesion (Rossi et al., 2019). Through the RGD region, MSC integrins bind to EPCs, contributing to the co-administration in angiogenesis, without hindering their differentiation (Rossi et al., 2017). Unfortunately, there is no definitive evidence as to why integrin serves in the recognition of MSCs and EPCs (Figure 2).

Overall, EPCs communicate with MSCs through multiple modes of action to achieve an efficient synergistic effect and promote bone regeneration. Compared with MSC treatment, EPCs aid local perfusion, inhibit early MSCs apoptosis, guide MSC differentiation, and enhance bone regeneration.

### Interaction of EPCs in Bone Vascularization

The bone marrow is dominated by microvasculature, capillaries of which are critical in the development, repair, and remodeling of the bone marrow for substance exchange and transport (Prisby, 2020). Capillaries mainly consist of a single layer of orderly placed ECs, acting as the intermediator between blood components and bone marrow. Additionally, SMCs form arterioles and venules around ECs, which should not be neglected. Simultaneously, EPCs directly form the elementary vascular plexus by indirect paracrine secretion of proangiogenic cytokines (Krenning et al., 2009). Such direct formation of vessels by EPCs is called vasculogenesis. In contrast, in angiogenesis, new vessels are formed on original vessels, and it is a key process in the revascularization of ischemic tissues and wound healing following birth (Masuda and Asahara, 2003). In addition to vasculogenesis guided by EPCs, other forms of vascularity are also present, and the interaction of EPCs among them should be explored.

EPCs differentiate into ECs and SMCs, and such differentiation is modulated by an array of factors (Zeng et al., 2021) (Table2) Differentiation into contractile- or synthetic-type SMCs by EPCs is observed by the addition of FDGF-BB and in the absence of endothelial cell growth factors (ECGF), which express a higher level of SMC markers compared with those expressed by mature ECs (Miyata et al., 2005). Moreover, the differentiation into contractile SMCs with ECGF deprivation is suppressed by the basic fibroblast growth factor, indicating its significant role in maintaining the phenotype of EPCs (Sai et al., 2014). Ehrba et al. modified and demonstrated the role of VEGF121 in the maturation of EPCs to ECs, in which their fibrin-bound variants caused a more efficacious maturation (Ehrbar et al., 2005). In addition, MSCs were also differentiated into SMCs, which are modulated by EPCs. With the use of the transwell co-culture system, gap junction inhibitor, and MEK inhibitor, EPCs promote MSCs to differentiate into SMCs both in cell-contact and ERK-dependent manners rather than the gap junction-dependent manner (Goerke et al., 2012). The effect of the secretion of CXCL12, CXCL1, VEGF, and macrophage migration inhibitory factor (MIF) by EPCs in a hypoxic environment on the differentiation is remarkable. Kanzler et al. observed that among the several secreted factors, MIF acted on the recruitment of cells that differentiated into an endothelial phenotype rather than CXCL12, CXCL1, or VEGF, by subcutaneous implantation of Matrigel. More importantly, MIF was almost the only factor to promote the differentiation of EPCs into SMCs. In contrast, CXCL12, despite being central in the recruitment of SMC progenitors, failed to stimulate and even restrained the differentiation of SMCs (Kucia et al., 2005; Kanzler et al., 2013).

**TABLE 2:** Modulation factors controlling EPCs differentiation into SMCs and ECs.

Cell type	*In vivo*/*in vitro*	Modulation factors	References
SMCs	*In vitro*	The addition of FDGF-BB and the absence of ECGF	Miyata et al. (2005)
	*In vitro*	The absence of bFGF	Sai et al. (2014)
	*In vivo* and *in vitro*	The addition of MIF rather than CXCL12	Kucia et al. (2005), Kanzler et al. (2013)
ECs	*In vitro*	VEGF121 and variants	Ehrbar et al. (2005)
	*In vivo* and *in vitro*	The secretion of MIF rather than CXCL12, CXCL1 or VEGF	Kucia et al. (2005), Kanzler et al. (2013)

EPCs and SMCs generate a favorable interplay and foster the development of functional neovessels. Smooth muscle progenitor cells (SMPCs) produce angiopoietin-1 (Ang-1), the angiogenic factor that further activates the receptor Tie-2 on EPCs, enabling improved EPC survival and stable formation of the vascular network (Foubert et al., 2008). Interestingly, Shudo et al. constructed a spatially oriented and chronologically sequenced SMC-EPC bi-level cell sheet in UpCell dishes, which retained the cell junctions and components of the extracellular matrix. Such cell sheets exert the release of SDF-1, VEGF, HGF, and TGF-β, which further amplify the upregulation of FLK1 and VEGFR2, indicating the potential cytokine communication (Shudo et al., 2013). Additional research reported their anti-fibrotic and anti-apoptotic properties since the expressions of TGF-β receptor, caspase-3, and caspase-9 decreased (Kawamura et al., 2017; Shudo et al., 2017). EPCs inhibited the TGF-β-induced pericyte transition *via* the paracrine pathway and the EPCs-MVs secretion, the mechanism of which is obscure (Yang et al., 2019). Likewise, Angiotensin (Ang) II-induced the transition of SMCs to synthetic phenotype, which is by EPC-EXOs. EPC-EXOs were consumed by caveolin-dependent endocytosis, delivering functional ACE2 and decreasing the activation of the NF-κB pathway (Wang J. et al., 2020).

In addition to differentiating into ECs, EPCs also directly regulate ECs during angiogenesis. EPCs promote the function of ECs by secreting a combination of growth factors directly. EPC-conditioned medium (EPC-CM) demonstrated the induction of EC maturation and angiogenic properties, both *in vivo* and *in vitro*, which further stimulated the recruitment of host EPCs (Di Santo et al., 2009; Maki et al., 2018). The proteome array of EPC secretome and other functional assays determined the effects of the proangiogenic factors, such as Ang, SDF-1, PDGF-BB, VEGF, and MMP, in vascular remodeling and EC proliferation (Maki et al., 2018). Furthermore, the addition of neomycin blocked the maturation of ECs, which inhibited the nuclear translocation of Ang for angiogenesis (Maki et al., 2018). In addition, EPCs-CM induced the activation of PI3K/AKT and MEK/ERK pathways in ECs and facilitated their functions in a time-dependent manner whereas the basal functions of ECs were not affected by the inhibition of pathways (Di Santo et al., 2014). Similarly, Yang et al. revealed effective cytoprotective properties of ECs through accommodation of intracellular antioxidant defense and pro-survival signals of paracrine factors of EPCs (Yang et al., 2010). The apoptosis of ECs was inhibited by the decreased expression of p53 upregulated modulator of apoptosis, a proapoptotic protein, and augmented expression of anti-apoptotic protein Bcl-2 in EPCs (Liang et al., 2015). Interestingly, Huang et al. detected that miR-1246 and miR-1290 in EPCs-EXOs provoked upregulation of E74-like factor five (ELF5) and Sp1 transcription factor (SP1), respectively, and enhanced phenotypic changes in ECs and angiogenesis both *in vivo* and *in vitro* (Huang Y. et al., 2021). EPC-MVs expressed certain adhesion molecules, such as intercellular adhesion molecule-1 (ICAM-1), α4 integrin, CD44, and CD29, which are essential for the internalization of MVs in ECs, and mRNA transport was especially crucial for the anti-apoptotic effect induced by MVs and for stimulating the organization of ECs. Moreover, MVs may initiate the activation of the PI3K/Akt signaling pathway and endothelial nitric oxide synthase (eNOS) in target ECs by enhancing the protein expression and phosphorylation of Akt and eNOS (Deregibus et al., 2007). Owing to interleukin (IL)-10 knockout, EPCs and EPC-EXOs left a detrimental impact on EC proliferation, tube formation, and enhanced apoptosis, through miR-375/PDK-1 signaling axis and NF-κB signaling with integrin-linked kinase enrichment in EXOs (Yue et al., 2020). EPC-EXOs mediators of paracrine signals completely inhibited hypoxia-reoxygenation-induced apoptotic and proinflammatory responses whereas microparticles and CM deprived of vesicles seemed effortless (Burger et al., 2015). Similarly, miR-21-5p was strongly affluent in EPC-EXOs and especially inhibited the expression of the angiogenesis inhibitor thrombospondin-1 in the recipient ECs, boosting repair (Hu et al., 2019). EPC-MVs shielded ECs from hypoxia-induced apoptosis by deregulating inflammatory and pro-apoptotic caspases and modulating elements engaged in mitochondrial and death receptor pathways (Deregibus et al., 2007).

Summing up, EPCs have a good synergistic relationship with SMCs and ECs, in terms of the enhanced function of single cells and intensive angiogenesis. EPCs differentiate into ECs and SMCs and secrete growth factors and EVs to regulate their functions, inhibit apoptosis, participate in neovasculature formation, and assist in bone regeneration.

### Interaction of EPCs in Bone Immunity

Additionally, the bone marrow is a lymphoid organ, from which a variety of immune cells stem and share the same bone marrow microenvironment, regulatory factors, and receptors as bone tissue. There is a complex interaction between bone cells and immune cells in both physiological and pathological states, including lymphocytes, dendritic cells, monocytes/macrophages (MMs), granulocytes, and mast cells. Unlike MSCs, which lack both MHC I and II, EPCs present higher levels of MHC II. Thus, EPCs have higher immunogenicity, as proved by their superior capacity to activate the proliferation of monocytes and CD8^+^ T cells *in vitro* (Tan K. et al., 2017)*.* Therefore, the interaction between EPCs and immune cells should be observed.

**FIGURE 3:**
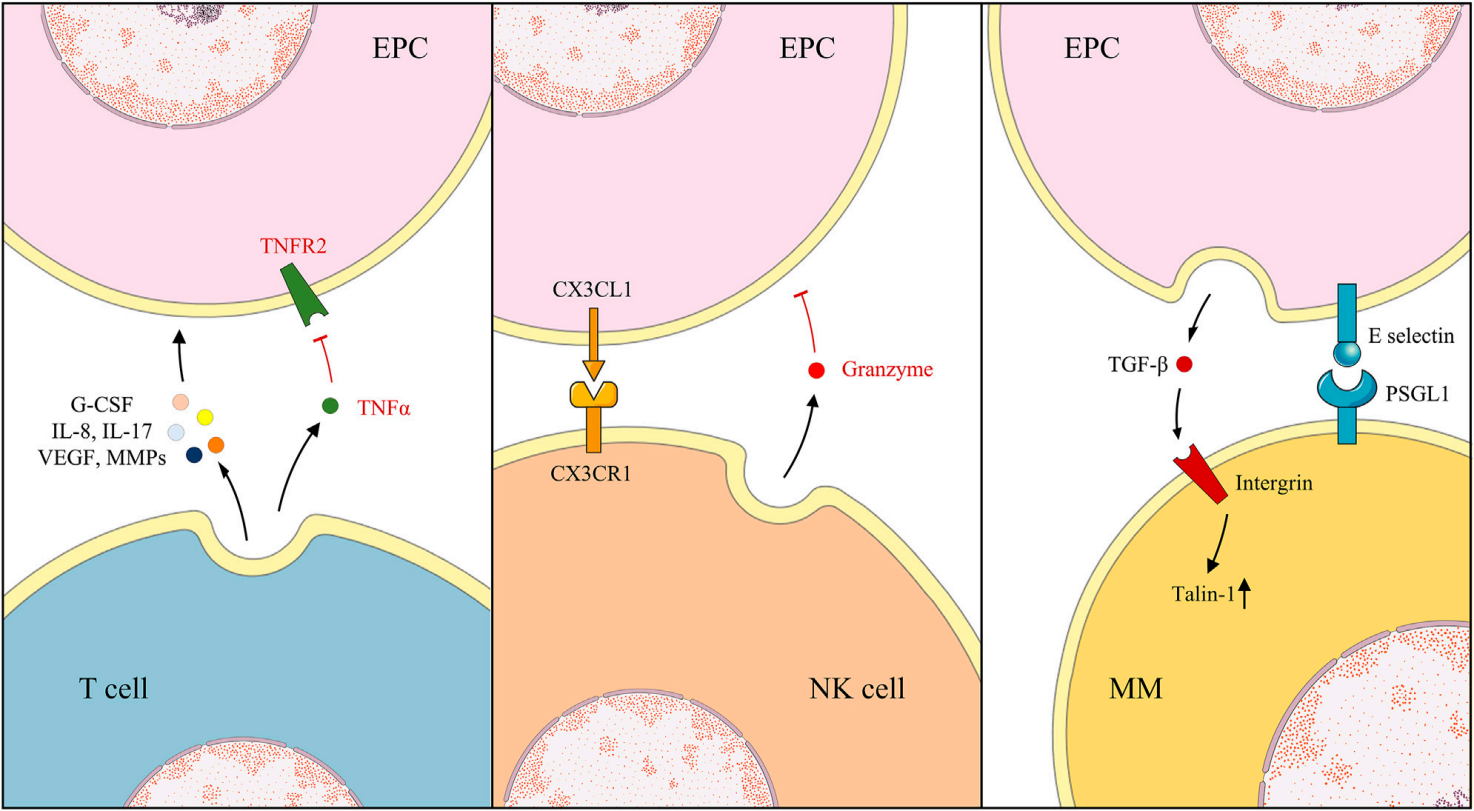
Under different environments, EPCs, T cells, NK cells, and MMs exhibit various action and reaction modes.

Under different circumstances, EPCs regulate the differentiation and infiltration of MMs. The EPC-CM reduced M1 MMs activation without changing M2 MMs and the expression of pro-inflammatory cytokines IL-1β and IL-6, alleviating inflammatory responses (Wang et al., 2018). By reducing ischemia/reperfusion injury-induced superoxide, the inflammatory agent macrophage inflammatory protein-2, and keratinocyte-derived cytokine production, EPCs may decrease MM infiltration, which was abundant in supply for superoxide, such as NADPH oxidase (Tojo et al., 2007; Burger et al., 2015; Liang et al., 2015). In the EPCs-MMs co-culture environment, the secretion of TGF-β1 from EPCs was detected, which binds to β integrins on the MMs surface, upregulating Talin-1 expression, activating downstream events, and causing the MMs migration and osteoclast differentiation (Cui et al., 2018). Additionally, the expressions of ICAM-1, vascular cell adhesion molecule 1, and E-selectin on the surface of EPCs were considered the mediators of the adhesion between EPCs and MMs (Shih et al., 2012). Chen et al. revealed that, during inflammation, expression of E-selectin on EPCs increased, causing increased adhesion to MMs and further adding to the inflammatory reaction and infiltration (Chen et al., 2018) (Figure 3)

The interaction between lymphocytes and EPCs is complex, and their regulation patterns and outcomes vary in different disease models. EPCs enhanced apoptosis, and hampered tube formation was observed *in vitro* when co-cultured with lymphocytes, even after adding angiogenic molecule hepatocyte growth factor (HGF) (Tan X. et al., 2017). By contrast, EPC tolerance by the host immune system and resistance in tissues were also testified in immunocompetent mice following several injections (Proust et al., 2020). EPCs suppressed T cells proliferation dose-dependently per the down-modulation of CD4^+^ and CD8^+^ T cells activation, causing a significant reduction in the secretion of TNF-α, interferon-γ, IL-2, and IL-17. The immunosuppressive effect relies on the TNF-α/TNF receptor 2 (TNFR2) pathway, as immunosuppression disappears in the absence of TNF-α from T cells or TNFR2 obstruction on the surface of EPCs (Naserian et al., 2020). This immunosuppressive effect of EPCs is enhanced in an inflammatory environment. With *LNK* gene knockout, implanted EPCs restrain the enrolment of cytotoxic T cells, macrophages, and neutrophils in the remodeling phase (Lee et al., 2016). Interestingly, Hur et al. observed that angiogenic T cells (Tang) promote vasculogenesis and endothelial repair by secreting high levels of angiogenic cytokines, such as VEGF, IL-8, IL-17, MMP, and granulocyte colony-stimulating factor (G-CSF). They constitute the center of EPC colonies and are essential in colony formation and differentiation of EPCs, depletion of which abrogated EPC functionality (Hur et al., 2007). In patients with rheumatoid arthritis, levels of Tang and EPCs consistently decreased and uniformly recovered following anti-TNF-α therapy (Rodríguez-Carrio et al., 2015a; Rodríguez-Carrio et al., 2015b). Interestingly, the regulation of EPCs and lymphocytes is also reflected in the correlation between EPCs and natural killer (NK) cells. NK cells augment the EPC lysis by the production of granzyme, a class of serine proteases mainly inducing pericellular death, and the recognition of CX3CL1 on EPCs by expressing CX3CR1. Nevertheless, in the EPCs-NK cell co-culture, a remarkable upregulation of N and E cadherin and VEGFR2 was detected in EPCs, indicating that NK cells enhanced angiogenesis, mechanisms of which remained obscure (Sehgal et al., 2020). In addition, no relevant studies on EPCs and B cells have been conducted; thus, it needs attention (Figure 3).

**FIGURE 4:**
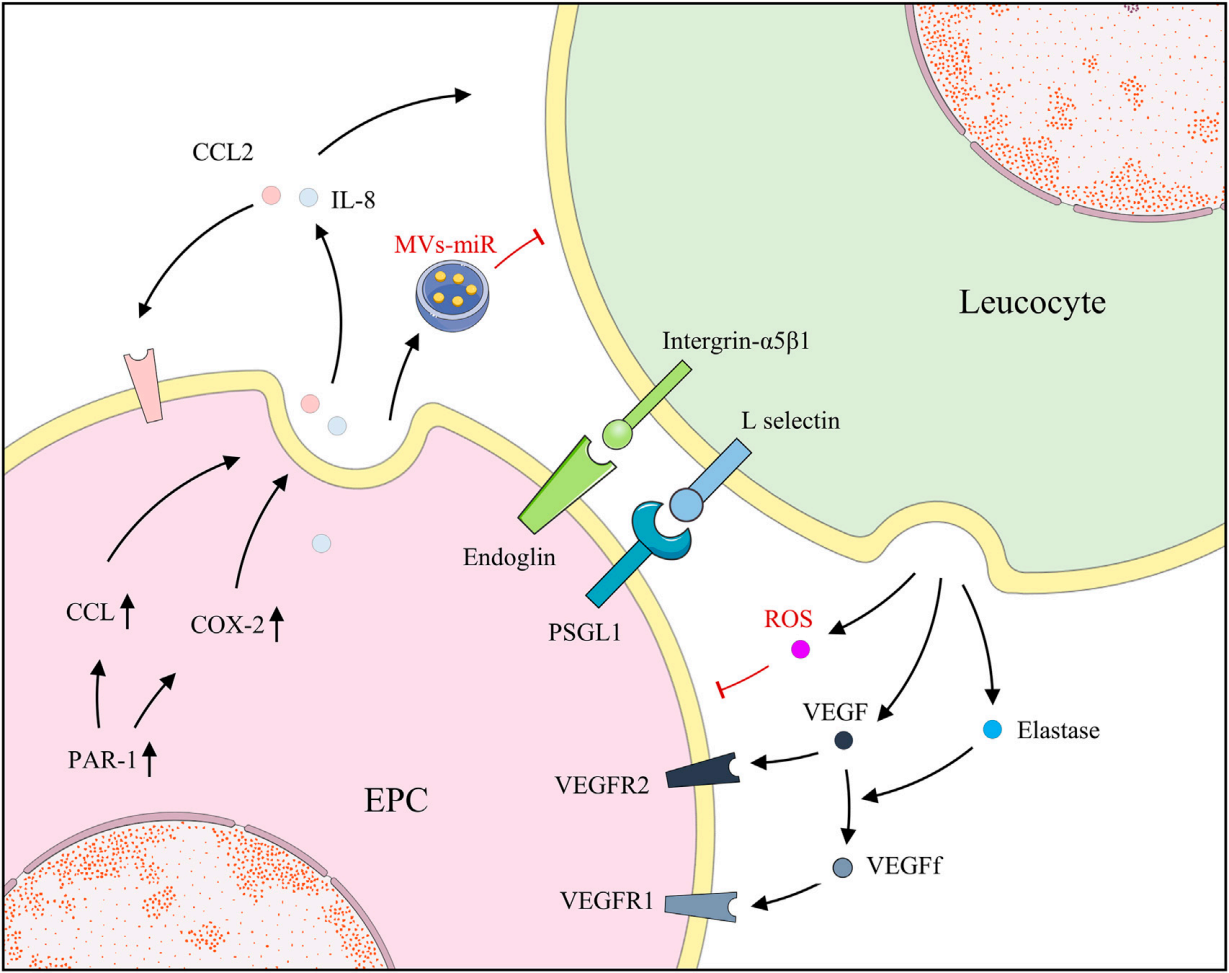
EPCs and leucocytes positively regulate each other by secreting factors; however, EPCs and leucocytes also secrete MVs and ROS, respectively, for negative regulation.

Generally, EPCs and neutrophils are beneficial but are detrimental to each other in some cases. Neutrophils facilitate angiogenesis, and previous studies have revealed that they activate and release MMP-2 and MMP-9 to aid in basement membrane degradation and contribute to angiogenesis (Muhs et al., 2004). G-CSF-activated neutrophils release VEGF, establishing an “angiogenic environment” to further promote EPC mobilization and local acquisition of vascular cells (Ohki et al., 2005). Moreover, the release and the function of elastase by leucocytes were determined, which targeted VEGF-A causing partial degradation to form a fragment of VEGF (VEGFf). Additionally, ECs migrated in response to integrated VEGF rather than VEGFf whereas MMs and EPCs were induced to migrate by either VEGF or VEGFf. VEGFf may enhance VEGF activity on ECs by inducing VEGFR1 through occupancy, thereby, reinforcing the interaction between integrated VEGF and VEGFR2 (Kurtagic et al., 2015). Neutrophils also communicate with EPCs by employing contact and adhesion. After binding with neutrophils, EPCs promptly presented the overexpression of ICAM-1, suggesting their potential recognition and mutual adjustment. Through the blocking of antibodies for EPCs and neutrophils by P-selectin glycoprotein ligand-1 (PSGL-1) and L-selectin, respectively, the accumulation of EPCs rather than neutrophils was repealed. However, the silencing of ICAM-1 on EPCs made no difference, which indicates that ligand was highly expressed on neutrophils similarly (Hubert et al., 2014). In contrast, Henrich et al. reported the bi-expression of CD18 and its counterpart, CD54, on EPCs and leucocytes, which caused the adhesion and the discharge of reactive oxygen species by leucocytes, impairing EPCs whereas neutrophil-derived elastase was considered negligible (Henrich et al., 2011). As previously reported, endoglin is widely distributed in the cell membranes of ECs and EPCs, especially at the site of leucocyte extravasation. Leucocytes adhere to the EPCs endoglin RGD motif *via* their integrin, α5β1, achieving activation and smooth transmigration (Rossi et al., 2013). With the activation of protease-activated receptor-1, expressions of cyclooxygenase-2 (COX-2) and CCL2 were revised in EPCs, observed in animals and clinical patients. COX-2, an important inflammatory pathway, further activated and released downstream IL-8, triggering the migration of neutrophils. Additionally, CCL2, a cellular chemokine important for the initiation and maintenance of inflammatory responses, is extracellularly released to recruit neutrophils and stimulate angiogenesis of EPCs, receptors of which are present (d'Audigier et al., 2015; Blandinières et al., 2019). Surprisingly, in multiple animal injury models, EPC transplantation notably attenuated the levels of several inflammatory factors and neutrophil infiltration, increasing levels of the anti-inflammatory cytokine IL-10 (Cao et al., 2012; Gao W. et al., 2019; Ju et al., 2019). Further experiments suggested that the key to the diminished neutrophil infiltration is the EPC-MVs, indicating that the embedded miRs are beneficial since RNase reduced the advantage (Cantaluppi et al., 2012) (Figure 4).

Overall, the modes of interaction and outcomes of EPCs and immune cells are complex and occasionally contradictory. This may be secondary to species, disease type, and state, albeit, unfortunately, we have not reached a definitive conclusion so far. Consequently, the immune regulation of the organism improves after treatment with EPCs, which promotes recovery from the disease. Thus, these contradictory results suggest a remarkable role of EPCs in immune regulation, which must be further explored.

## Application of EPCs in Bone Biology

### EPCs in Osteoporosis

Osteoporosis is a bone disease featuring a bone strength regression and an increased fracture threat. It is usually asymptomatic or associated with mild symptoms and is not only a common cause of clinically pathological fractures but also one of the high-risk factors affecting human health. Meanwhile, with the widespread use of steroids, the prevalence of osteoporosis is high not only in elderly patients but also in younger patients (Xi et al., 2020). Mature EPCs are positively correlated with bone mass and angiogenesis- or osteogenesis-related cytokines in bone tissue in comparison between patients with osteoporosis and people with normal bone marrow, which confirmed the previous findings of EPCs mediating the interaction between angiogenesis and osteogenesis. Additionally, senile osteoporosis indicated decreased EPCs numbers and impaired maturation, which may provide perspectives for osteoporosis mechanisms and treatments (Cheng et al., 2018). In an animal model of steroid-induced osteoporosis, the volume and density of bone trabeculae and marrow increased owing to EPC-EVs therapy; and through further bioinformatics analysis, EPC-EV treatment partially inhibited the iron death pathway in osteoblasts and reversed steroid-induced oxidative damage (Lu et al., 2019). Li et al. supported the notion of Wnt3a signaling, which involves the function of the spinal load on stimulation of osteoblast differentiation and promotion of EPCs migration and tube formation in ovariectomized mice model of osteoporosis (Li et al., 2019). Carrying pH-responsive nanoparticles, which target bone in mice model of osteoporosis, promotes EPCs vascularization since preosteoclasts continually generate PDGF-BB, activating focal adhesion kinase by PI3K-Akt (Dou et al., 2021). Previous CD34^+^ cells were considered to contain EPCs, which enhanced osteoblasts and simultaneously impaired the activity of osteoclasts for osteoporosis treatment (Aggarwal et al., 2012).

### EPCs in Bone Fracture and Defect

Bone fracture and defect are common traumatic bone diseases, severely damaging vasculature and disrupting circulation in the injured area, which may contribute to the threat of inefficient healing. Bone fracture healing and defect regeneration are complex processes affected by many factors, including inflammatory responses and angiogenesis. Previous studies have revealed that neovascularization during the early stages of fracture healing is regulated by the mobilization of bone marrow-derived EPCs to the fracture site *via* peripheral circulation (Matsumoto et al., 2008). The ratio of EPCs among the peripheral blood increased immediately following bone fracture and returned to basal lines during recovery (Lee et al., 2008). EPCs mobilizing cytokines and homing molecules were upregulated at the fracture callus, such as VEGF, monocyte chemoattractant protein-1, and SDF-1 (Lee et al., 2008). Matsumoto et al. hypothesized and certified the curative potency of circulating CD34^+^ cells, contributing to an environment favorable to angiogenesis and osteogenesis and thus, completely healing the fracture (Matsumoto et al., 2006). Additionally, after quality and quantity control culture, CD34^+^ cells increased and exhibited markedly better angiogenic potential and higher bone union rate in monocytes (Mifuji et al., 2017). Furthermore, with G-CSF-mobilized CD34^+^ cells loaded on atelocollagen scaffolds, nonunion fractures mostly exhibited radiographs of fracture healing in the clinical trial (Matsumoto et al., 2006). Li et al. transferred EPCs to the bone defect, which elevated BMP-2 expression compared with the control group (Li et al., 2014). Consistent with the work of Li et al., EPC transplantation increased neovascularization and BMP-2 gene edition in MSCs, and EPCs significantly promoted bone regeneration (He et al., 2013). Moreover, grafted EPCs released VEGF to recruit host EPCs and induced angiogenesis in the bone defect, which is an important indirect effect (Li et al., 2020). EPCs accelerated bone fracture healing and regeneration *via* the SDF-1/CXCR4 axis, an essential interaction in vascular development (Zhang R. et al., 2019).

Nonetheless, there are few available options to promote angiogenesis in artificial bone grafts, excluding exogenous EPCs grafts, clinical application of which is hampered by the source, security, expense, and time. Thus, considerable efforts were done to recruit, capture, and maintain EPCs on synthetic scaffolds (Zhuang et al., 2021). By immobilizing bioactive peptides on scaffolds, dynamic recruitment of EPCs was observed, which, thereafter, supported initial angiogenesis and eventual osteogenesis (Li et al., 2020). Similarly, by upregulating the CXCR4 pathway, osteoprotegerin enhanced the proliferation and migration of EPCs, both of which accelerated angiogenesis and osteogenesis in bone defect areas (Zhang R. et al., 2019).

### EPCs in Distraction Osteogenesis

Distraction osteogenesis (DO) is a new endogenous tissue engineering technique, an effective treatment for bone defects, bone hypoplasia, and craniofacial deformities, with the advantage of eliminating the need for exogenous implants. The process of DO includes intraoperative truncation, retractor placement, postoperative internal fixation, and slow traction osteogenesis during the distraction period. Although DO has good efficacy, its prolonged fixation and complication risk limit its clinical use (Jiang et al., 2021). Thus, the mechanism of action and the prospect of the application of EPCs in DO must be studied. Doppler flow analysis revealed relative ischemia during the initial phase in the DO, and the EPC population exhibited a significant growth at the ischemic site during the activation phase and retained consolidated (Cetrulo et al., 2005). Lee et al. evaluated EPC colony-forming units after isolating and culturing MNCs in patients undergoing limb lengthening surgery. EPC-enriched cell fractions in freshly isolated MNCs significantly increased during the distraction period, and EPC-mobilizing factors VEGF and SDF-1 significantly increased in plasma (Lee et al., 2010). Furthermore, Fujio et al. constructed a high-speed DO (H-DO) model, in which the distraction was double the speed of normal DO, and observed deficient callus regeneration in the distraction gap, secondary to ineffective recruitment of EPCs/ECs. They tested and confirmed the ability of local affixation of SDF-1 in H-DO, which successfully induced callus formation by recruiting and maturing EPCs/ECs, neo-blood vessels maturation *via* enrolling α-SMA + pericytes, and smooth blood circulation (Fujio et al., 2011). Jia et al. directly injected EPC-EXOs, with EPCs as a positive control, into the distraction gap, which exerted the stimulation of angiogenesis during DO. They further observed the proangiogenic effects of EPC-EXOs based on miR-126, which was predominantly concentrated in EPC-EXOs and targeted SPRED-1, inhibiting reticular activating system/ERK signaling by hindering the Raf activation (Jia et al., 2019).

## Challenges and Prospects

Since their discovery, EPCs have been reported to have a remarkable contribution to the development and treatment of several diseases. They aid in neovascularization and further impact bone regeneration by interacting with diversified cells (Masuda and Asahara, 2003).

We have not yet completely studied the role of EPCs in the regulation of osteoclastogenesis, the direction of differentiation, and impacting blood flow, which should be further explored.

Studies on the role of EPCs in bone tissue have mostly explored their effects on osteogenesis, which is insufficient. Therapeutic strategies in bone fracture models have observed that EPCs-EXO regulate miR-124 levels *via* LncRNA-MALAT1 to augment recruitment and differentiation of osteoclast precursors, thereby, aiding bone restoration *in vivo* (Cui et al., 2019). In contrast with the detection of osteoclast-related markers in the EPCs-hydroxyapatite poly scaffolds, there were no indications of increased osteoclast-like activity (Shi et al., 2016). These results are insufficient to explain the role of EPCs in the regulation of osteoclastogenesis and can even draw completely contrasting conclusions. Tanaka et al. observed that CM promoted angiogenesis *in vitro* for osteoclasts and the osteoclast-derived angiogenic activity was terminated using neutralizing antibodies on osteopontin (Tanaka et al., 2007). They also revealed that osteoclasts stimulate the migration and survival of human umbilical vein endothelial cells (HUVECs) and osteopontin and VEGF induce the release of soluble osteoclastogenic factors from HUVECs (Tanaka et al., 2007). Additionally, osteoclasts secrete Ang, which preserves the proliferative activity of ECs through plexin-B2-mediated transcription of ribosomal RNA and promotes angiogenesis (Liu et al., 2021). Osteoclast precursors generated PDGF-BB to facilitate the development of type H vessels, which subsequently stimulated osteoblastogenesis (Xie et al., 2014). Taken together, osteoclasts positively impact angiogenesis and promote the function of ECs by secreting a considerable amount of growth factors. Although studies on the interaction between EPCs and osteoclasts are rare, can we compare the interaction between EPCs and osteoclasts and speculate significant mutual promotion of vascularization and osteogenesis during bone regeneration? Simultaneously, the dynamic balance between osteoclasts and osteoblasts is of great importance in bone physiology and pathology (Feng and Teitelbaum, 2013). Our knowledge of the regulation of osteogenesis and osteoclastogenesis by EPCs is one-sided, and no relevant studies are exploring the effect of the addition of EPCs in the dynamic balance of osteogenesis and osteoclastogenesis in normal and abnormal states, especially during bone regeneration. Based on the studies we have described and summarized, we hypothesize that EPCs enhance the function of osteoblasts during bone regeneration and regulate or even inhibit the effect of osteoclasts. They may even possibly modulate the infiltration and differentiation of immune cells, such as MMs, during bone regeneration and affect osteoclastogenesis by promoting the differentiation of MMs into M2 MMs rather than osteoclasts.

In addition, studies on the types of angiogenesis, involving EPCs, have not been reported so far. Recent studies have revealed that there are two subtypes of ECs distributed in capillaries of bone tissue in mice and humans, which are divided into type H and type L vessels based on differences in surface antibody expression (Kusumbe et al., 2014). Although ECs of subtype H were minor, a large number of Osterix + osteoprogenitor cells, collagen-like 1α+ osteoblasts, and Runx2+ early osteoprogenitor cells were clustered around type H vessels whereas almost no osteoprogenitor cells were distributed around type L vessels (Saran et al., 2014). Concurrently, a series of studies has revealed that paracrine mechanisms involving multiple cells in the bone marrow microenvironment are essential for the formation of type H vessels and osteogenesis. As previously described, PDGF BB, which is mainly secreted by pro-osteoclasts in the bone marrow and peripheral blood, maintains the periosteal microenvironment and supports osteogenesis and formation of type H vessels by upregulating periostin expression and triggering PI3K/AKT cascade to recruit MSCs, EPCs, and periosteum-derived cells (Xie et al., 2014; Gao B. et al., 2019). Furthermore, the depletion of PDGF BB and the preferential association of Osterix + osteoprogenitor cells and type H vessels were significantly low, especially in the transcortical lamina and osteogenic fronts (Rindone et al., 2021). Mature osteoblasts and osteoclasts also secrete slit guidance ligand 3 (SLIT3) and display a remarkable decrease in subtype HECs, reduced bone mass, diminished osteogenic activity, and enhanced osteolysis in its absence (Kim et al., 2018; Xu et al., 2018). Interestingly, HIF-1α is also essential for type H vessels and exhibits a significant aging-dependent effect, which eventually diminishes or even disappears (Kusumbe et al., 2014). Although NOTCH signaling inhibited ECs proliferation and angiogenesis in other organs, its inverse effect was observed in bone. In response to NOTCH signaling, ECs of subtype H exhibited markedly enhanced proliferation and high expression of Noggin protein. Correspondingly, higher vascular flow in type H vessels boosts NOTCH signaling (Ramasamy et al., 2014; Ramasamy et al., 2016). Noggin, an antagonist of BMP, modulates osteogenesis *in vivo*, normalizes the number of osteoprogenitor cells, restores the organization of the bone vascular system, and increases the expression of VEGF-A (Ramasamy et al., 2014). Meanwhile, type H vessels were confirmed in the alveolar bone and tooth extraction socket. ECs of subtype H and Runx2+ osteoprogenitor cells were detected and accumulated at the restoration stage, indicating the potential benefits of ECs of subtype H in bone regeneration (Yan et al., 2020). Additionally, increased formation of type H vessels, consistent with enhanced bone healing, was observed during the treatment of fractures, whether by supplementation with recombinant SLIT3 or by low-intensity pulsed ultrasound (Xu et al., 2016; Xu et al., 2018). The information about the promotion of bone tissue regeneration by EPCs is currently based on their strong angiogenic capacity and the functional enhancement of various types of cells generated by the abundant vascular network (Kim et al., 2021). However, information about the type of blood vessels formed by their differentiation and the related regulatory mechanisms is not known. Based on the efficient synergistic effect of EPCs and MSCs, we boldly propose the hypothesis that EPCs differentiate mainly into ECs of subtype H and recruit and induce a large number of osteoblasts, thus, accelerating bone regeneration.

Similarly, blood flow controls vascular features and osteogenesis (Ramasamy et al., 2016). Blood flow affects vascular stability and morphology by adjusting the proliferation of ECs and the recruitment of mural cells *via* the flow receptors of ECs (Baeyens et al., 2016). In the physiological state, in response to higher blood flow and shear stress, higher activity of the Notch pathway in ECs and strengthened angiogenesis and osteogenesis were confirmed (Ramasamy et al., 2016). Moreover, in bone fracture murine models, the blood flow in the fractured area decreased below the baseline level on the first day, peaked gradually, and further exhibited a general decrease with fluctuations. Additionally, group comparisons revealed that the earlier the blood flow peaked, the faster and more effectively the fracture healed (Ren et al., 2020). In the bone graft healing model, the autograft group presented a peak in the blood flow in the first week, a decrease by half in the second week, and a steady decrease thereafter. The allograft group seeded with MSCs on hydrogel exhibited a similar peak but with more discrepancy in decline (Han et al., 2016). Although, to date, there have been no relevant studies on the hemodynamics of blood vessel formation during osteogenesis by EPCs. However, relevant studies have been reporting the functional changes of the vessels formed by EPCs in different states, which provide us with some guidelines. In the hypoxic microenvironment, EPCs are involved in the formation of immature neovascularization with enlarged lumen, disorganized branching, increased instability, and susceptibility to rupture (Kashiwazaki et al., 2018). EPCs-EV treatment significantly improved hemodynamics and vascular structure and enhanced cardiac function following myocardial infarction (Chung et al., 2020). Generally, bone tissue is sensitive to mechanical stimuli, and its hemodynamics determined by vascular morphology is particularly important. Furthermore, based on previous studies, we observed that EPCs have more angiogenic effects and mediate poor neovascular morphology and unstable blood flow in the pathological state (Kashiwazaki et al., 2018). Accordingly, we speculate that favorable EPCs positively impact bone regeneration at an early stage, owing to not only the generation of a more extensive vascular network but also that of a more stable and ordered vascular network, which promotes bone regeneration under the dual effect of material transport and mechanical signal stimulation.

## Conclusion

In this review, we reported and summarized the functions and interactions of EPCs in bone. EPCs interact effectively with various cells in a communicative manner, creating a powerful synergistic effect. Through paracrine and pro-secretory EVs and intercellular junctions, EPCs secrete growth factors or directly regulate the function of the remaining cells and are moderated correspondingly. Despite many limitations, the mechanism of the action of EPCs in bone biology should be further explored, and thus, EPCs should be used as one of the potential strategies for the treatment of bone diseases.

## Glossary

ac-LDL: acetylated low-density lipoprotein
Tang: angiogenic T cells
Ang: angiopoietin
RGD: arginine-glycine-aspartic acid
bFGF: basic fibroblast growth factor
BMP: bone morphogenetic protein
CM: conditioned medium
CXCR2: C-X-C chemokine receptor 2
COX: cycloxygenase
ELF5: e74-like factor five
ECGF: endothelial cell growth factors
ECs: endothelial cells
ENOS: endothelial nitric oxide synthase
EPCs: endothelial progenitor cells
ECFCs: endothelial colony-forming cells
EXOs: exosomes
EVs: extracellular vesicles
VEGFf: fragment of VEGF
G-CSF: granulocyte colony-stimulating factor
HGF: hepatocyte growth factor
IGF: Insulin-like growth factor
ICAM: intercellular adhesion molecule
IFN-γ: Interferon-γ
IL: interleukin
KC: keratinocyte-derived cytokine
PDGF: platelet-derived growth factor
MIP: macrophage inflammatory protein
MIF: macrophage migration inhibitory factor
MMP: matrix metalloproteinases
MSCs: mesenchymal stem cells
MP: microparticle
miR: microRNA
MVs: microvesicles
MCP: monocyte chemoattractant protein
MMs: monocytes/macrophages
MACs: myeloid angiogenic cells
NK: natural killer
THBS1: thrombospondin-1
PUMA: p53 upregulated modulator of apoptosis
PAR: protease-activated receptor
PSGL: p-selectin glycoprotein ligand
ROS: reactive oxygen species
SLIT3: slit guidance ligand 3
SMCs: smooth muscle cells
sKitL
soluble kit-ligand SP1: SP1sp1 transcription factor
SDF: stromal cell-derived factor
TGF-β: transforming growth factor β
TNF-α: tumor necrosis factor-α
UEA: *Ulex europaeus* agglutinin.

## References

[B1] Aggarwal R. Lu J. Kanji S. Joseph M. Das M. Noble G. J. (2012). Human Umbilical Cord Blood-Derived CD34+ Cells Reverse Osteoporosis in NOD/SCID Mice by Altering Osteoblastic and Osteoclastic Activities. PLoS One 7, e39365. 10.1371/journal.pone.0039365 22724005 PMC3377665

[B2] Asahara T. Murohara T. Sullivan A. Silver M. van der Zee R. Li T. (1997). Isolation of Putative Progenitor Endothelial Cells for Angiogenesis. Science 275, 964–966. 10.1126/science.275.5302.964 9020076

[B3] Baeyens N. Larrivée B. Ola R. Hayward-Piatkowskyi B. Dubrac A. Huang B. (2016). Defective Fluid Shear Stress Mechanotransduction Mediates Hereditary Hemorrhagic Telangiectasia. J. Cel Biol 214, 807–816. 10.1083/jcb.201603106 PMC5037412 27646277

[B4] Blandinières A. Gendron N. Bacha N. Bièche I. Chocron R. Nunes H. (2019). Interleukin-8 Release by Endothelial colony-forming Cells Isolated from Idiopathic Pulmonary Fibrosis Patients Might Contribute to Their Pathogenicity. Angiogenesis 22, 325–339. 10.1007/s10456-018-09659-5 30607696

[B5] Bouland C. Philippart P. Dequanter D. Corrillon F. Loeb I. Bron D. (2021). Cross-Talk between Mesenchymal Stromal Cells (MSCs) and Endothelial Progenitor Cells (EPCs) in Bone Regeneration. Front. Cel Dev. Biol. 9, 674084. 10.3389/fcell.2021.674084 PMC8166285 34079804

[B6] Burger D. Viñas J. L. Akbari S. Dehak H. Knoll W. Gutsol A. (2015). Human Endothelial Colony-Forming Cells Protect against Acute Kidney Injury. Am. J. Pathol. 185, 2309–2323. 10.1016/j.ajpath.2015.04.010 26073035

[B7] Cantaluppi V. Gatti S. Medica D. Figliolini F. Bruno S. Deregibus M. C. (2012). Microvesicles Derived from Endothelial Progenitor Cells Protect the Kidney from Ischemia-Reperfusion Injury by microRNA-dependent Reprogramming of Resident Renal Cells. Kidney Int. 82, 412–427. 10.1038/ki.2012.105 22495296

[B8] Cao J.-P. He X.-Y. Xu H.-T. Zou Z. Shi X.-Y. (2012). Autologous Transplantation of Peripheral Blood-Derived Circulating Endothelial Progenitor Cells Attenuates Endotoxin-Induced Acute Lung Injury in Rabbits by Direct Endothelial Repair and Indirect Immunomodulation. Anesthesiology 116, 1278–1287. 10.1097/aln.0b013e3182567f84 22546965

[B9] Cetrulo C. L. JR. Knox K. R. Brown D. J. Ashinoff R. L. Dobryansky M. Ceradini D. J. (2005). Stem Cells and Distraction Osteogenesis: Endothelial Progenitor Cells home to the Ischemic Generate in Activation and Consolidation. Plast. Reconstr. Surg. 116, 1053–1064. discussion 1065-7. 10.1097/01.prs.0000178403.79051.70 16163094

[B10] Chambers S. E. J. O'Neill C. L. Guduric-Fuchs J. Mcloughlin K. J. Liew A. Egan A. M. (2018). The Vasoreparative Function of Myeloid Angiogenic Cells Is Impaired in Diabetes through the Induction of IL1β. Stem Cells 36, 834–843. 10.1002/stem.2810 29484768 PMC6001623

[B11] Chen G. Li P. Liu Z. Zeng R. Ma X. Chen Y. (2019). Enrichment of miR-126 Enhances the Effects of Endothelial Progenitor Cell-Derived Microvesicles on Modulating MC3T3-E1 Cell Function via Erk1/2-Bcl-2 Signalling Pathway. Prion 13, 106–115. 10.1080/19336896.2019.1607464 31050590 PMC7000145

[B12] Chen W. Xiao L. Bai J. Zeng W. Yang M. Shi B. (2018). The Promotion of Tissue Engineering Blood Vessel Patency by CGS21680 through Regulating Pro‐inflammatory Activities of Endothelial Progenitor Cell. J. Biomed. Mater. Res. 106, 2634–2642. 10.1002/jbm.a.36457 29790247

[B13] Cheng Q. Lin S. Bi B. Jiang X. Shi H. Fan Y. (2018). Bone Marrow-Derived Endothelial Progenitor Cells Are Associated with Bone Mass and Strength. J. Rheumatol. 45, 1696–1704. 10.3899/jrheum.171226 30173148

[B14] Chung J. J. Han J. Wang L. L. Arisi M. F. Zaman S. Gordon J. (2020). Delayed Delivery of Endothelial Progenitor Cell-Derived Extracellular Vesicles via Shear Thinning Gel Improves Postinfarct Hemodynamics. J. Thorac. Cardiovasc. Surg. 159, 1825–1835. e2. 10.1016/j.jtcvs.2019.06.017 31353103 PMC7077034

[B15] Cui Y. Fu S. Hou T. Wu X. (2018). Endothelial Progenitor Cells Enhance the Migration and Osteoclastic Differentiation of Bone Marrow-Derived Macrophages *In Vitro* and in a Mouse Femur Fracture Model through Talin-1. Cell Physiol Biochem 49, 555–564. 10.1159/000492993 30165361

[B16] Cui Y. Fu S. Sun D. Xing J. Hou T. Wu X. (2019). EPC‐derived Exosomes Promote Osteoclastogenesis through LncRNA‐MALAT1. J. Cel Mol Med 23, 3843–3854. 10.1111/jcmm.14228 PMC6533478 31025509

[B17] d'Audigier C. Cochain C. Rossi E. Guérin C. L. Bièche I. Blandinières A. (2015). Thrombin Receptor PAR-1 Activation on Endothelial Progenitor Cells Enhances Chemotaxis-Associated Genes Expression and Leukocyte Recruitment by a COX-2-dependent Mechanism. Angiogenesis 18, 347–359. 10.1007/s10456-015-9471-8 26026674

[B18] Deregibus M. C. Cantaluppi V. Calogero R. Lo Iacono M. Tetta C. Biancone L. (2007). Endothelial Progenitor Cell-Derived Microvesicles Activate an Angiogenic Program in Endothelial Cells by a Horizontal Transfer of mRNA. Blood 110, 2440–2448. 10.1182/blood-2007-03-078709 17536014

[B19] Di Santo S. Seiler S. Fuchs A.-L. Staudigl J. Widmer H. R. (2014). The Secretome of Endothelial Progenitor Cells Promotes Brain Endothelial Cell Activity through PI3-Kinase and MAP-Kinase. PLoS One 9, e95731. 10.1371/journal.pone.0095731 24755675 PMC3995762

[B20] Di Santo S. Yang Z. Wyler von Ballmoos M. Voelzmann J. Diehm N. Baumgartner I. (2009). Novel Cell-free Strategy for Therapeutic Angiogenesis: *In Vitro* Generated Conditioned Medium Can Replace Progenitor Cell Transplantation. PLoS One 4, e5643. 10.1371/journal.pone.0005643 19479066 PMC2682571

[B21] Dou C. Li J. He J. Luo F. Yu T. Dai Q. (2021). Bone-targeted pH-Responsive Cerium Nanoparticles for Anabolic Therapy in Osteoporosis. Bioactive Mater. 6, 4697–4706. 10.1016/j.bioactmat.2021.04.038 PMC8164008 34095626

[B22] Ehrbar M. Metters A. Zammaretti P. Hubbell J. A. Zisch A. H. (2005). Endothelial Cell Proliferation and Progenitor Maturation by Fibrin-Bound VEGF Variants with Differential Susceptibilities to Local Cellular Activity. J. Controlled Release 101, 93–109. 10.1016/j.jconrel.2004.07.018 15588897

[B23] Feng X. Teitelbaum S. L. (2013). Osteoclasts: New Insights. Bone Res. 1, 11–26. 10.4248/BR201301003 26273491 PMC4472093

[B24] Foubert P. Matrone G. Souttou B. Leré-Déan C. Barateau V. Plouët J. (2008). Coadministration of Endothelial and Smooth Muscle Progenitor Cells Enhances the Efficiency of Proangiogenic Cell-Based Therapy. Circ. Res. 103, 751–760. 10.1161/circresaha.108.175083 18723447

[B25] Franz S. Rammelt S. Scharnweber D. Simon J. C. (2011). Immune Responses to Implants - a Review of the Implications for the Design of Immunomodulatory Biomaterials. Biomaterials 32, 6692–6709. 10.1016/j.biomaterials.2011.05.078 21715002

[B26] Fujio M. Yamamoto A. Ando Y. Shohara R. Kinoshita K. Kaneko T. (2011). Stromal Cell-Derived Factor-1 Enhances Distraction Osteogenesis-Mediated Skeletal Tissue Regeneration through the Recruitment of Endothelial Precursors. Bone 49, 693–700. 10.1016/j.bone.2011.06.024 21741502

[B27] Gao B. Deng R. Chai Y. Chen H. Hu B. Wang X. (2019). Macrophage-lineage TRAP+ Cells Recruit Periosteum-Derived Cells for Periosteal Osteogenesis and Regeneration. J. Clin. Invest. 129, 2578–2594. 10.1172/jci98857 30946695 PMC6538344

[B28] Gao W. Jiang T. Liu Y.-h. Ding W.-g. Guo C.-c. Cui X.-g. (2019). Endothelial Progenitor Cells Attenuate the Lung Ischemia/reperfusion Injury Following Lung Transplantation via the Endothelial Nitric Oxide Synthase Pathway. J. Thorac. Cardiovasc. Surg. 157, 803–814. 10.1016/j.jtcvs.2018.08.092 30391008

[B29] George A. L. Bangalore-Prakash P. Rajoria S. Suriano R. Shanmugam A. Mittelman A. (2011). Endothelial Progenitor Cell Biology in Disease and Tissue Regeneration. J. Hematol. Oncol. 4, 24. 10.1186/1756-8722-4-24 21609465 PMC3123653

[B30] Goerke S. M. Plaha J. Hager S. Strassburg S. Torio-Padron N. Stark G. B. (2012). Human Endothelial Progenitor Cells Induce Extracellular Signal-Regulated Kinase-dependent Differentiation of Mesenchymal Stem Cells into Smooth Muscle Cells upon Cocultivation. Tissue Eng. A 18, 2395–2405. 10.1089/ten.tea.2012.0147 22731749

[B31] Grosso A. Burger M. G. Lunger A. Schaefer D. J. Banfi A. di Maggio N. (2017). It Takes Two to Tango: Coupling of Angiogenesis and Osteogenesis for Bone Regeneration. Front. Bioeng. Biotechnol. 5, 68. 10.3389/fbioe.2017.00068 29164110 PMC5675838

[B32] Han S. Proctor A. R. Vella J. B. Benoit D. S. W. Choe R. (2016). Non-invasive Diffuse Correlation Tomography Reveals Spatial and Temporal Blood Flow Differences in Murine Bone Grafting Approaches. Biomed. Opt. Express 7, 3262–3279. 10.1364/boe.7.003262 27699097 PMC5030009

[B33] Hattori K. Heissig B. Tashiro K. Honjo T. Tateno M. Shieh J.-H. (2001). Plasma Elevation of Stromal Cell-Derived Factor-1 Induces Mobilization of Mature and Immature Hematopoietic Progenitor and Stem Cells. Blood 97, 3354–3360. 10.1182/blood.v97.11.3354 11369624

[B34] He X. Dziak R. Yuan X. Mao K. Genco R. Swihart M. (2013). BMP2 Genetically Engineered MSCs and EPCs Promote Vascularized Bone Regeneration in Rat Critical-Sized Calvarial Bone Defects. PLoS One 8, e60473. 10.1371/journal.pone.0060473 23565253 PMC3614944

[B35] Heissig B. Hattori K. Dias S. Friedrich M. Ferris B. Hackett N. R. (2002). Recruitment of Stem and Progenitor Cells from the Bone Marrow Niche Requires MMP-9 Mediated Release of Kit-Ligand. Cell 109, 625–637. 10.1016/s0092-8674(02)00754-7 12062105 PMC2826110

[B36] Henrich D. Zimmer S. Seebach C. Frank J. Barker J. Marzi I. (2011). Trauma-Activated Polymorphonucleated Leukocytes Damage Endothelial Progenitor Cells. Shock 36, 216–222. 10.1097/shk.0b013e3182236eba 21610569

[B37] Hu H. Wang B. Jiang C. Li R. Zhao J. (2019). Endothelial Progenitor Cell-Derived Exosomes Facilitate Vascular Endothelial Cell Repair through Shuttling miR-21-5p to Modulate Thrombospondin-1 Expression. Clin. Sci. (Lond) 133, 1629–1644. 10.1042/cs20190188 31315970

[B38] Huang Y. Chen L. Feng Z. Chen W. Yan S. Yang R. (2021). EPC-derived Exosomal miR-1246 and miR-1290 Regulate Phenotypic Changes of Fibroblasts to Endothelial Cells to Exert Protective Effects on Myocardial Infarction by Targeting ELF5 and SP1. Front. Cel Dev. Biol. 9, 647763. 10.3389/fcell.2021.647763 PMC8155602 34055778

[B39] Huang Z. Liu Z. Wang K. Ye Z. Xiong Y. Zhang B. (2021). Reduced Number and Activity of Circulating Endothelial Progenitor Cells in Acute Aortic Dissection and its Relationship with IL-6 and IL-17. Front. Cardiovasc. Med. 8, 628462. 10.3389/fcvm.2021.628462 33869300 PMC8044799

[B40] Hubert L. Darbousset R. Panicot-Dubois L. Robert S. Sabatier F. Fallague K. (2014). Neutrophils Recruit and Activate Human Endothelial colony-forming Cells at the Site of Vessel Injury via P-Selectin Glycoprotein Ligand-1 and L-Selectin. J. Thromb. Haemost. 12, 1170–1181. 10.1111/jth.12551 24606340

[B41] Hur J. Yang H.-M. Yoon C.-H. Lee C.-S. Park K.-W. Kim J.-H. (2007). Identification of a Novel Role of T Cells in Postnatal Vasculogenesis. Circulation 116, 1671–1682. 10.1161/circulationaha.107.694778 17909106

[B42] Jia Y. Zhu Y. Qiu S. Xu J. Chai Y. (2019). Exosomes Secreted by Endothelial Progenitor Cells Accelerate Bone Regeneration during Distraction Osteogenesis by Stimulating Angiogenesis. Stem Cel Res Ther 10, 12. 10.1186/s13287-018-1115-7 PMC6329174 30635031

[B43] Jiang W. Zhu P. Zhang T. Liao F. Yu Y. Liu Y. (2021). MicroRNA-205 Mediates Endothelial Progenitor Functions in Distraction Osteogenesis by Targeting the Transcription Regulator NOTCH2. Stem Cel Res Ther 12, 101. 10.1186/s13287-021-02150-x PMC7860583 33536058

[B44] Joo H. J. Song S. Seo H.-R. Shin J. H. Choi S.-C. Park J. H. (2015). Human Endothelial colony Forming Cells from Adult Peripheral Blood Have Enhanced Sprouting Angiogenic Potential through Up-Regulating VEGFR2 Signaling. Int. J. Cardiol. 197, 33–43. 10.1016/j.ijcard.2015.06.013 26113473

[B45] Ju Y.-n. Geng Y.-j. Wang X.-t. Gong J. Zhu J. Gao W. (2019). Endothelial Progenitor Cells Attenuate Ventilator-Induced Lung Injury with Large-Volume Ventilation. Cel Transpl. 28, 1674–1685. 10.1177/0963689719874048 PMC6923558 31526054

[B46] Kamprom W. Kheolamai P. U-Pratya A. Wattanapanitch M. Laowtammathron C. Roytrakul S (2016b). Endothelial Progenitor Cell Migration-Enhancing Factors in the Secretome of Placental-Derived Mesenchymal Stem Cells. Stem Cell Int 2016, 2514326. 10.1155/2016/2514326 PMC4736766 26880942

[B47] Kamprom W. Kheolamai P. U-Pratya Y. Supokawej A. Wattanapanitch M. Laowtammathron C. (2016a). Effects of Mesenchymal Stem Cell-Derived Cytokines on the Functional Properties of Endothelial Progenitor Cells. Eur. J. Cel Biol. 95, 153–163. 10.1016/j.ejcb.2016.02.001 26899034

[B48] Kanzler I. Tuchscheerer N. Steffens G. Simsekyilmaz S. Konschalla S. Kroh A. (2013). Differential Roles of Angiogenic Chemokines in Endothelial Progenitor Cell-Induced Angiogenesis. Basic Res. Cardiol. 108, 310. 10.1007/s00395-012-0310-4 23184390 PMC3786139

[B49] Kashiwazaki D. Koh M. Uchino H. Akioka N. Kuwayama N. Noguchi K. (2018). Hypoxia Accelerates Intraplaque Neovascularization Derived from Endothelial Progenitor Cells in Carotid Stenosis. J. Neurosurg. 131, 884–891. 10.3171/2018.4.JNS172876 30485214

[B50] Kawamura M. Paulsen M. J. Goldstone A. B. Shudo Y. Wang H. Steele A. N. (2017). Tissue-engineered Smooth Muscle Cell and Endothelial Progenitor Cell Bi-level Cell Sheets Prevent Progression of Cardiac Dysfunction, Microvascular Dysfunction, and Interstitial Fibrosis in a Rodent Model of Type 1 Diabetes-Induced Cardiomyopathy. Cardiovasc. Diabetol. 16, 142. 10.1186/s12933-017-0625-4 29096622 PMC5668999

[B51] Keshavarz S. Nassiri S. M. Siavashi V. Alimi N. S. (2019). Regulation of Plasticity and Biological Features of Endothelial Progenitor Cells by MSC-Derived SDF-1. Biochim. Biophys. Acta (Bba) - Mol. Cel Res. 1866, 296–304. 10.1016/j.bbamcr.2018.11.013 30502369

[B52] Kim B.-J. Lee Y.-S. Lee S.-Y. Baek W.-Y. Choi Y. J. Moon S. A. (2018). Osteoclast-secreted SLIT3 Coordinates Bone Resorption and Formation. J. Clin. Invest. 128, 1429–1441. 10.1172/jci91086 29504949 PMC5873876

[B53] Kim H. D. Hong X. An Y. H. Park M. J. Kim D. G. Greene A. K. (2021). A Biphasic Osteovascular Biomimetic Scaffold for Rapid and Self-Sustained Endochondral Ossification. Adv. Healthc. Mater. 10, e2100070. 10.1002/adhm.202100070 33882194 PMC8273143

[B54] Krenning G. van Luyn M. J. A. Harmsen M. C. (2009). Endothelial Progenitor Cell-Based Neovascularization: Implications for Therapy. Trends Mol. Med. 15, 180–189. 10.1016/j.molmed.2009.02.001 19303359

[B55] Kucia M. Reca R. Miekus K. Wanzeck J. Wojakowski W. Janowska‐Wieczorek A. (2005). Trafficking of Normal Stem Cells and Metastasis of Cancer Stem Cells Involve Similar Mechanisms: Pivotal Role of the SDF‐1-CXCR4 Axis. Stem Cells 23, 879–894. 10.1634/stemcells.2004-0342 15888687

[B56] Kurtagic E. Rich C. B. Buczek-Thomas J. A. Nugent M. A. (2015). Neutrophil Elastase-Generated Fragment of Vascular Endothelial Growth Factor-A Stimulates Macrophage and Endothelial Progenitor Cell Migration. PLoS One 10, e0145115. 10.1371/journal.pone.0145115 26672607 PMC4682631

[B57] Kusumbe A. P. Ramasamy S. K. Adams R. H. (2014). Coupling of Angiogenesis and Osteogenesis by a Specific Vessel Subtype in Bone. Nature 507, 323–328. 10.1038/nature13145 24646994 PMC4943525

[B58] Lee D. Y. Cho T.-J. Kim J. A. Lee H. R. Yoo W. J. Chung C. Y. (2008). Mobilization of Endothelial Progenitor Cells in Fracture Healing and Distraction Osteogenesis. Bone 42, 932–941. 10.1016/j.bone.2008.01.007 18326482

[B59] Lee D. Y. Cho T.-J. Lee H. R. Park M. S. Yoo W. J. Chung C. Y. (2010). Distraction Osteogenesis Induces Endothelial Progenitor Cell Mobilization without Inflammatory Response in Man. Bone 46, 673–679. 10.1016/j.bone.2009.10.018 19853677

[B60] Lee J. H. Ji S. T. Kim J. Takaki S. Asahara T. Hong Y.-J. (2016). Specific Disruption of Lnk in Murine Endothelial Progenitor Cells Promotes Dermal Wound Healing via Enhanced Vasculogenesis, Activation of Myofibroblasts, and Suppression of Inflammatory Cell Recruitment. Stem Cel Res Ther 7, 158. 10.1186/s13287-016-0403-3 PMC5084514 27793180

[B61] Li A. Cheng X. J. Moro A. Singh R. K. Hines O. J. Eibl G. (2011). CXCR2-Dependent Endothelial Progenitor Cell Mobilization in Pancreatic Cancer Growth. Translational Oncol. 4, 20–28. 10.1593/tlo.10184 PMC3026405 21286374

[B62] Li L. Liu W. Zhao Y. Ma P. Zha S. Chen P. (2020). Dual-Peptide-Functionalized Nanofibrous Scaffolds Recruit Host Endothelial Progenitor Cells for Vasculogenesis to Repair Calvarial Defects. ACS Appl. Mater. Inter. 12, 3474–3493. 10.1021/acsami.9b21434 31874023

[B63] Li L. Zhou G. Wang Y. Yang G. Ding S. Zhou S. (2015). Controlled Dual Delivery of BMP-2 and Dexamethasone by Nanoparticle-Embedded Electrospun Nanofibers for the Efficient Repair of Critical-Sized Rat Calvarial Defect. Biomaterials 37, 218–229. 10.1016/j.biomaterials.2014.10.015 25453952

[B64] Li R. Nauth A. Gandhi R. Syed K. Schemitsch E. H. (2014). BMP-2 mRNA Expression after Endothelial Progenitor Cell Therapy for Fracture Healing. J. Orthop. Trauma 28 Suppl 1 (Suppl. 1), S24–S27. 10.1097/BOT.0000000000000071 24464097

[B65] Li X. Liu D. Li J. Yang S. Xu J. Yokota H. (2019). Wnt3a Involved in the Mechanical Loading on Improvement of Bone Remodeling and Angiogenesis in a Postmenopausal Osteoporosis Mouse Model. FASEB j. 33, 8913–8924. 10.1096/fj.201802711r 31017804 PMC9272758

[B66] Li Z. Yang A. Yin X. Dong S. Luo F. Dou C. (2018). Mesenchymal Stem Cells Promote Endothelial Progenitor Cell Migration, Vascularization, and Bone Repair in Tissue‐engineered Constructs via Activating CXCR2‐Src‐PKL/Vav2‐Rac1. FASEB j. 32, 2197–2211. 10.1096/fj.201700895r 29229683

[B67] Liang C.-J. Shen W.-C. Chang F.-B. Wu V.-C. Wang S.-H. Young G.-H. (2015). Endothelial Progenitor Cells Derived from Wharton's Jelly of Human Umbilical Cord Attenuate Ischemic Acute Kidney Injury by Increasing Vascularization and Decreasing Apoptosis, Inflammation, and Fibrosis. Cel Transpl. 24, 1363–1377. 10.3727/096368914x681720 24819279

[B68] Lin R.-Z. Moreno-Luna R. Li D. Jaminet S.-C. Greene A. K. Melero-Martin J. M. (2014). Human Endothelial colony-forming Cells Serve as Trophic Mediators for Mesenchymal Stem Cell Engraftment via Paracrine Signaling. Proc. Natl. Acad. Sci. U.S.A. 111, 10137–10142. 10.1073/pnas.1405388111 24982174 PMC4104912

[B69] Liu X. Chai Y. Liu G. Su W. Guo Q. Lv X. (2021). Osteoclasts Protect Bone Blood Vessels against Senescence through the Angiogenin/plexin-B2 axis. Nat. Commun. 12, 1832. 10.1038/s41467-021-22131-1 33758201 PMC7987975

[B70] Lu J. Yang J. Zheng Y. Chen X. Fang S. (2019). Extracellular Vesicles from Endothelial Progenitor Cells Prevent Steroid-Induced Osteoporosis by Suppressing the Ferroptotic Pathway in Mouse Osteoblasts Based on Bioinformatics Evidence. Sci. Rep. 9, 16130. 10.1038/s41598-019-52513-x 31695092 PMC6834614

[B71] Maes C. Kobayashi T. Selig M. K. Torrekens S. Roth S. I. Mackem S. (2010). Osteoblast Precursors, but Not Mature Osteoblasts, Move into Developing and Fractured Bones along with Invading Blood Vessels. Develop. Cel 19, 329–344. 10.1016/j.devcel.2010.07.010 PMC3540406 20708594

[B72] Maki T. Morancho A. Martinez-San Segundo P. Hayakawa K. Takase H. Liang A. C. (2018). Endothelial Progenitor Cell Secretome and Oligovascular Repair in a Mouse Model of Prolonged Cerebral Hypoperfusion. Stroke 49, 1003–1010. 10.1161/strokeaha.117.019346 29511131 PMC5871569

[B73] Masuda H. Asahara T. (2003). Post-natal Endothelial Progenitor Cells for Neovascularization in Tissue Regeneration. Cardiovasc. Res. 58, 390–398. 10.1016/s0008-6363(02)00785-x 12757873

[B74] Matsumoto T. Kawamoto A. Kuroda R. Ishikawa M. Mifune Y. Iwasaki H. (2006). Therapeutic Potential of Vasculogenesis and Osteogenesis Promoted by Peripheral Blood CD34-Positive Cells for Functional Bone Healing. Am. J. Pathol. 169, 1440–1457. 10.2353/ajpath.2006.060064 17003498 PMC1698844

[B75] Matsumoto T. Mifune Y. Kawamoto A. Kuroda R. Shoji T. Iwasaki H. (2008). Fracture Induced Mobilization and Incorporation of Bone Marrow-Derived Endothelial Progenitor Cells for Bone Healing. J. Cel. Physiol. 215, 234–242. 10.1002/jcp.21309 18205179

[B76] Medina R. J. Barber C. L. Sabatier F. Dignat-George F. Melero-Martin J. M. Khosrotehrani K. (2017). Endothelial Progenitors: A Consensus Statement on Nomenclature. Stem Cell Transl Med 6, 1316–1320. 10.1002/sctm.16-0360 PMC5442722 28296182

[B77] Mifuji K. Ishikawa M. Kamei N. Tanaka R. Arita K. Mizuno H. (2017). Angiogenic Conditioning of Peripheral Blood Mononuclear Cells Promotes Fracture Healing. Bone Jt. Res. 6, 489–498. 10.1302/2046-3758.68.bjr-2016-0338.r1 PMC5579315 28835445

[B78] Miyata T. Iizasa H. Sai Y. Fujii J. Terasaki T. Nakashima E. (2005). Platelet-derived Growth Factor-BB (PDGF-BB) Induces Differentiation of Bone Marrow Endothelial Progenitor Cell-Derived Cell Line TR-BME2 into Mural Cells and Changes the Phenotype. J. Cel. Physiol. 204, 948–955. 10.1002/jcp.20362 15828021

[B79] Muhs B. E. Gagne P. Plitas G. Shaw J. P. Shamamian P. (2004). Experimental Hindlimb Ischemia Leads to Neutrophil-Mediated Increases in Gastrocnemius MMP-2 and -9 Activity: a Potential Mechanism for Ischemia Induced MMP Activation. J. Surg. Res. 117, 249–254. 10.1016/j.jss.2003.09.009 15047130

[B80] Murphy K. C. Stilhano R. S. Mitra D. Zhou D. Batarni S. Silva E. A. (2016). Hydrogel Biophysical Properties Instruct Coculture‐mediated Osteogenic Potential. FASEB j. 30, 477–486. 10.1096/fj.15-279984 26443826 PMC4684517

[B81] Naserian S. Abdelgawad M. E. Afshar Bakshloo M. Ha G. Arouche N. Cohen J. L. (2020). The TNF/TNFR2 Signaling Pathway Is a Key Regulatory Factor in Endothelial Progenitor Cell Immunosuppressive Effect. Cell Commun Signal 18, 94. 10.1186/s12964-020-00564-3 32546175 PMC7298859

[B82] Ohki Y. Heissig B. Sato Y. Akiyama H. Zhu Z. Hicklin D. J. (2005). Granulocyte colony‐stimulating Factor Promotes Neovascularization by Releasing Vascular Endothelial Growth Factor from Neutrophils. FASEB j. 19, 2005–2007. 10.1096/fj.04-3496fje 16223785

[B83] Pang H. Wu X.-H. Fu S.-L. Luo F. Zhang Z.-H. Hou T.-Y. (2013). Prevascularisation with Endothelial Progenitor Cells Improved Restoration of the Architectural and Functional Properties of Newly Formed Bone for Bone Reconstruction. Int. Orthopaedics (Sicot) 37, 753–759. 10.1007/s00264-012-1751-y PMC3609986 23288045

[B84] Percival C. J. Richtsmeier J. T. (2013). Angiogenesis and Intramembranous Osteogenesis. Dev. Dyn. 242, 909–922. 10.1002/dvdy.23992 23737393 PMC3803110

[B85] Premer C. Wanschel A. Porras V. Balkan W. Legendre-Hyldig T. Saltzman R. G. (2019). Mesenchymal Stem Cell Secretion of SDF-1α Modulates Endothelial Function in Dilated Cardiomyopathy. Front. Physiol. 10, 1182. 10.3389/fphys.2019.01182 31616309 PMC6769040

[B86] Prisby R. D. (2020). Bone Marrow Microvasculature. Compr. Physiol. 10, 1009–1046. 10.1002/cphy.c190009 32941689

[B87] Proust R. Ponsen A.-C. Rouffiac V. Schenowitz C. Montespan F. Ser-le Roux K. (2020). Cord Blood-Endothelial colony Forming Cells Are Immunotolerated and Participate at postischemic Angiogenesis in an Original Dorsal Chamber Immunocompetent Mouse Model. Stem Cel Res Ther 11, 172. 10.1186/s13287-020-01687-7 PMC7206734 32381102

[B88] Quarto R. Mastrogiacomo M. Cancedda R. Kutepov S. M. Mukhachev V. Lavroukov A. (2001). Repair of Large Bone Defects with the Use of Autologous Bone Marrow Stromal Cells. N. Engl. J. Med. 344, 385–386. 10.1056/nejm200102013440516 11195802

[B89] Ramasamy S. K. Kusumbe A. P. Schiller M. Zeuschner D. Bixel M. G. Milia C. (2016). Blood Flow Controls Bone Vascular Function and Osteogenesis. Nat. Commun. 7, 13601. 10.1038/ncomms13601 27922003 PMC5150650

[B90] Ramasamy S. K. Kusumbe A. P. Wang L. Adams R. H. (2014). Endothelial Notch Activity Promotes Angiogenesis and Osteogenesis in Bone. Nature 507, 376–380. 10.1038/nature13146 24647000 PMC4943529

[B91] Ren J. Han S. Proctor A. R. Desa D. E. Ramirez G. A. Ching‐Roa V. R. D. (2020). Longitudinal 3D Blood Flow Distribution provided by Diffuse Correlation Tomography during Bone Healing in a Murine Fracture Model. Photochem. Photobiol. 96, 380–387. 10.1111/php.13201 31883385 PMC7138748

[B92] Rindone A. N. Liu X. Farhat S. Perdomo-Pantoja A. Witham T. F. Coutu D. L. (2021). Quantitative 3D Imaging of the Cranial Microvascular Environment at Single-Cell Resolution. Nat. Commun. 12, 6219. 10.1038/s41467-021-26455-w 34711819 PMC8553857

[B93] Rodríguez-Carrio J. Alperi-LóPEZ M. LóPEZ P. Alonso-Castro S. Ballina-GARCíA F. J. SUáREZ A. (2015a). Angiogenic T Cells Are Decreased in Rheumatoid Arthritis Patients. Ann. Rheum. Dis. 74, 921–927. 10.1136/annrheumdis-2013-204250 24399233

[B94] Rodríguez-Carrio J. Alperi-LóPEZ M. LóPEZ P. Ballina-GARCíA F. J. SUáREZ A. (2015b). Good Response to Tumour Necrosis Factor Alpha Blockade Results in an Angiogenic T Cell Recovery in Rheumatoid Arthritis Patients. Rheumatology (Oxford) 54, 1129–1131. 10.1093/rheumatology/kev025 25832609

[B95] Rossi E. Bernabeu C. Smadja D. M. (2019). Endoglin as an Adhesion Molecule in Mature and Progenitor Endothelial Cells: A Function beyond TGF-β. Front. Med. 6, 10. 10.3389/fmed.2019.00010 PMC6363663 30761306

[B96] Rossi E. Goyard C. Cras A. Dizier B. Bacha N. Lokajczyk A. (2017). Coinjection of Mesenchymal Stem Cells with Endothelial Progenitor Cells Accelerates Muscle Recovery in Hind Limb Ischemia through an Endoglin-dependent Mechanism. Thromb. Haemost. 117, 1908–1918. 10.1160/th17-01-0007 28771278

[B97] Rossi E. Sanz-Rodriguez F. Eleno N. Düwell A. Blanco F. J. Langa C. (2013). Endothelial Endoglin Is Involved in Inflammation: Role in Leukocyte Adhesion and Transmigration. Blood 121, 403–415. 10.1182/blood-2012-06-435347 23074273

[B98] Sai Y. Nishimura T. Muta M. Iizasa H. Miyata T. Nakashima E. (2014). Basic Fibroblast Growth Factor Is Essential to Maintain Endothelial Progenitor Cell Phenotype in TR-BME2 Cells. Biol. Pharm. Bull. 37, 688–693. 10.1248/bpb.b13-00841 24694617

[B99] Saleh F. A. Whyte M. Ashton P. Genever P. G. (2011). Regulation of Mesenchymal Stem Cell Activity by Endothelial Cells. Stem Cell Develop. 20, 391–403. 10.1089/scd.2010.0168 20536359

[B100] Saran U. Gemini Piperni S. Chatterjee S. (2014). Role of Angiogenesis in Bone Repair. Arch. Biochem. Biophys. 561, 109–117. 10.1016/j.abb.2014.07.006 25034215

[B101] Schlundt C. El Khassawna T. Serra A. Dienelt A. Wendler S. Schell H. (2018). Macrophages in Bone Fracture Healing: Their Essential Role in Endochondral Ossification. Bone 106, 78–89. 10.1016/j.bone.2015.10.019 26529389

[B102] Seebach C. Henrich D. Kähling C. Wilhelm K. Tami A. E. Alini M. (2010). Endothelial Progenitor Cells and Mesenchymal Stem Cells Seeded onto β-TCP Granules Enhance Early Vascularization and Bone Healing in a Critical-Sized Bone Defect in Rats. Tissue Eng. Part A 16, 1961–1970. 10.1089/ten.tea.2009.0715 20088701

[B103] Seebach E. Freischmidt H. Holschbach J. Fellenberg J. Richter W. (2014). Mesenchymal Stroma Cells Trigger Early Attraction of M1 Macrophages and Endothelial Cells into Fibrin Hydrogels, Stimulating Long Bone Healing without Long-Term Engraftment. Acta Biomater. 10, 4730–4741. 10.1016/j.actbio.2014.07.017 25058402

[B104] Sehgal R. Kaur S. Shasthry S. M. Agrawal T. Dwivedi V. Seth D. (2020). Natural Killer Cells Contribute to Pathogenesis of Severe Alcoholic Hepatitis by Inducing Lysis of Endothelial Progenitor Cells. Alcohol. Clin. Exp. Res. 44, 78–86. 10.1111/acer.14242 31746472

[B105] Shi Y. Wang F. Tiwari S. Yesilbas M. Steubesand N. Weitkamp J.-T. (2016). Role of Myeloid Early Endothelial Progenitor Cells in Bone Formation and Osteoclast Differentiation in Tissue Construct Based on Hydroxyapatite Poly(ester-Urethane) Scaffolds. J. Orthop. Res. 34, 1922–1932. 10.1002/jor.23222 26945676

[B106] Shih Y.-T. Wang M.-C. Peng H.-H. Chen T.-F. Chen L. Chang J.-Y. (2012). Modulation of Chemotactic and Pro-inflammatory Activities of Endothelial Progenitor Cells by Hepatocellular Carcinoma. Cell Signal. 24, 779–793. 10.1016/j.cellsig.2011.11.013 22120522

[B107] Shudo Y. Cohen J. E. Macarthur J. W. Atluri P. Hsiao P. F. Yang E. C. (2013). Spatially Oriented, Temporally Sequential Smooth Muscle Cell-Endothelial Progenitor Cell Bi-level Cell Sheet Neovascularizes Ischemic Myocardium. Circulation 128, S59–S68. 10.1161/CIRCULATIONAHA.112.000293 24030422 PMC4111240

[B108] Shudo Y. Goldstone A. B. Cohen J. E. Patel J. B. Hopkins M. S. Steele A. N. (2017). Layered Smooth Muscle Cell-Endothelial Progenitor Cell Sheets Derived from the Bone Marrow Augment Postinfarction Ventricular Function. J. Thorac. Cardiovasc. Surg. 154, 955–963. 10.1016/j.jtcvs.2017.04.081 28651946 PMC5947323

[B109] Tamari T. Kawar-Jaraisy R. Doppelt O. Giladi B. Sabbah N. Zigdon-Giladi H. (2020). The Paracrine Role of Endothelial Cells in Bone Formation via CXCR4/SDF-1 Pathway. Cells 9,1325. 10.3390/cells906132510.3390/cells9061325 PMC7349013 32466427

[B110] Tan K. Zheng K. Li D. Lu H. Wang S. Sun X. (2017a). Impact of Adipose Tissue or Umbilical Cord Derived Mesenchymal Stem Cells on the Immunogenicity of Human Cord Blood-Derived Endothelial Progenitor Cells. PLoS One 12, e0178624. 10.1371/journal.pone.0178624 28562647 PMC5451078

[B111] Tan X. Juan F.-g. Shah A. Q. (2017b). Involvement of Endothelial Progenitor Cells in the Formation of Plexiform Lesions in Broiler Chickens: Possible Role of Local Immune/inflammatory Response. J. Zhejiang Univ. Sci. B 18, 59–69. 10.1631/jzus.b1600500 28070997 PMC5260478

[B112] Tanaka Y. Abe M. Hiasa M. Oda A. Amou H. Nakano A. (2007). Myeloma Cell-Osteoclast Interaction Enhances Angiogenesis Together with Bone Resorption: a Role for Vascular Endothelial Cell Growth Factor and Osteopontin. Clin. Cancer Res. 13, 816–823. 10.1158/1078-0432.ccr-06-2258 17289872

[B113] Tasev D. Koolwijk P. van Hinsbergh V. W. M. (2016). Therapeutic Potential of Human-Derived Endothelial Colony-Forming Cells in Animal Models. Tissue Eng. B: Rev. 22, 371–382. 10.1089/ten.teb.2016.0050 27032435

[B114] Tojo A. Asaba K. Onozato M. L. (2007). Suppressing Renal NADPH Oxidase to Treat Diabetic Nephropathy. Expert Opin. Ther. Targets 11, 1011–1018. 10.1517/14728222.11.8.1011 17665974

[B115] Walter D. H. Haendeler J. Reinhold J. Rochwalsky U. Seeger F. Honold J. (2005). Impaired CXCR4 Signaling Contributes to the Reduced Neovascularization Capacity of Endothelial Progenitor Cells from Patients with Coronary Artery Disease. Circ. Res. 97, 1142–1151. 10.1161/01.res.0000193596.94936.2c 16254213

[B116] Wang J. Li J. Cheng C. Liu S. (2020). Angiotensin-converting Enzyme 2 Augments the Effects of Endothelial Progenitor Cells-Exosomes on Vascular Smooth Muscle Cell Phenotype Transition. Cell Tissue Res 382, 509–518. 10.1007/s00441-020-03259-w 32852610

[B117] Wang L. Wei J. Da Fonseca Ferreira A. Wang H. Zhang L. Zhang Q. (2020). Rejuvenation of Senescent Endothelial Progenitor Cells by Extracellular Vesicles Derived from Mesenchymal Stromal Cells. JACC: Basic Translational Sci. 5, 1127–1141. 10.1016/j.jacbts.2020.08.005 PMC7691285 33294742

[B118] Wang S. Aurora A. B. Johnson B. A. Qi X. Mcanally J. Hill J. A. (2008). The Endothelial-specific microRNA miR-126 Governs Vascular Integrity and Angiogenesis. Develop. Cel 15, 261–271. 10.1016/j.devcel.2008.07.002 PMC2685763 18694565

[B119] Wang T. Fang X. Yin Z. S. (2018). Endothelial Progenitor Cell-Conditioned Medium Promotes Angiogenesis and Is Neuroprotective after Spinal Cord Injury. Neural Regen. Res. 13, 887–895. 10.4103/1673-5374.232484 29863020 PMC5998635

[B120] Watson L. Elliman S. J. Coleman C. M. (2014). From Isolation to Implantation: a Concise Review of Mesenchymal Stem Cell Therapy in Bone Fracture Repair. Stem Cel Res Ther 5, 51. 10.1186/scrt439 PMC4055164 25099622

[B121] Wen L. Wang Y. Wen N. Yuan G. Wen M. Zhang L. (2016). Role of Endothelial Progenitor Cells in Maintaining Stemness and Enhancing Differentiation of Mesenchymal Stem Cells by Indirect Cell-Cell Interaction. Stem Cell Develop. 25, 123–138. 10.1089/scd.2015.0049 26528828

[B122] Wu P. Zhang B. Ocansey D. K. W. Xu W. Qian H. (2021). Extracellular Vesicles: A Bright star of Nanomedicine. Biomaterials 269, 120467. 10.1016/j.biomaterials.2020.120467 33189359

[B123] Xi L. de Falco P. Barbieri E. Karunaratne A. Bentley L. Esapa C. T. (2020). Reduction of Fibrillar Strain-Rate Sensitivity in Steroid-Induced Osteoporosis Linked to Changes in Mineralized Fibrillar Nanostructure. Bone 131, 115111. 10.1016/j.bone.2019.115111 31726107

[B124] Xie H. Cui Z. Wang L. Xia Z. Hu Y. Xian L. (2014). PDGF-BB Secreted by Preosteoclasts Induces Angiogenesis during Coupling with Osteogenesis. Nat. Med. 20, 1270–1278. 10.1038/nm.3668 25282358 PMC4224644

[B125] Xu C. Liu H. He Y. Li Y. He X. (2020). Endothelial Progenitor Cells Promote Osteogenic Differentiation in Cocultured with Mesenchymal Stem Cells via the MAPK-dependent Pathway. Stem Cel Res Ther 11, 537. 10.1186/s13287-020-02056-0 PMC7731475 33308309

[B126] Xu R. Yallowitz A. Qin A. Wu Z. Shin D. Y. Kim J.-M. (2018). Targeting Skeletal Endothelium to Ameliorate Bone Loss. Nat. Med. 24, 823–833. 10.1038/s41591-018-0020-z 29785024 PMC5992080

[B127] Xu X. Wang F. Yang Y. Zhou X. Cheng Y. Wei X. (2016). LIPUS Promotes Spinal Fusion Coupling Proliferation of Type H Microvessels in Bone. Sci. Rep. 6, 20116. 10.1038/srep20116 26830666 PMC4735589

[B128] Yan Z. Q. Wang X.-K. Zhou Y. Wang Z. G. Wang Z. X. Jin L. (2020). H‐type Blood Vessels Participate in Alveolar Bone Remodeling during Murine Tooth Extraction Healing. Oral Dis. 26, 998–1009. 10.1111/odi.13321 32144839

[B129] Yang J. Wang M. Zhu F. Sun J. Xu H. Chong Lee Shin O. L.-S. (2019). Putative Endothelial Progenitor Cells Do Not Promote Vascular Repair but Attenuate Pericyte-Myofibroblast Transition in UUO-Induced Renal Fibrosis. Stem Cel Res Ther 10, 104. 10.1186/s13287-019-1201-5 PMC6429829 30898157

[B130] Yang Z. von Ballmoos M. W. Faessler D. Voelzmann J. Ortmann J. Diehm N. (2010). Paracrine Factors Secreted by Endothelial Progenitor Cells Prevent Oxidative Stress-Induced Apoptosis of Mature Endothelial Cells. Atherosclerosis 211, 103–109. 10.1016/j.atherosclerosis.2010.02.022 20227693

[B131] Yue Y. Wang C. Benedict C. Huang G. Truongcao M. Roy R. (2020). Interleukin-10 Deficiency Alters Endothelial Progenitor Cell-Derived Exosome Reparative Effect on Myocardial Repair via Integrin-Linked Kinase Enrichment. Circ. Res. 126, 315–329. 10.1161/circresaha.119.315829 31815595 PMC7015105

[B132] Zeng W. Lei Q. Ma J. Gao S. Ju R. (2021). Endothelial Progenitor Cell-Derived Microvesicles Promote Angiogenesis in Rat Brain Microvascular Endothelial Cells *In Vitro* . Front. Cel. Neurosci. 15, 638351. 10.3389/fncel.2021.638351 PMC7930325 33679329

[B133] Zhang K.-L. Wang Y.-J. Sun J. Zhou J. Xing C. Huang G. (2019). Artificial Chimeric Exosomes for Antiphagocytosis and Targeted Cancer Therapy. Chem. Sci. 10, 1555–1561. 10.1039/c8sc03224f 30809374 PMC6357862

[B134] Zhang R. Liu J. Yu S. Sun D. Wang X. Fu J. (2019). Osteoprotegerin (OPG) Promotes Recruitment of Endothelial Progenitor Cells (EPCs) via CXCR4 Signaling Pathway to Improve Bone Defect Repair. Med. Sci. Monit. 25, 5572–5579. 10.12659/msm.916838 31350844 PMC6681686

[B135] Zhang Y. Xie Y. Hao Z. Zhou P. Wang P. Fang S. (2021). Umbilical Mesenchymal Stem Cell-Derived Exosome-Encapsulated Hydrogels Accelerate Bone Repair by Enhancing Angiogenesis. ACS Appl. Mater. Inter. 13, 18472–18487. 10.1021/acsami.0c22671 33856781

[B136] Zhuang Y. Zhang C. Cheng M. Huang J. Liu Q. Yuan G. (2021). Challenges and Strategies for *In Situ* Endothelialization and Long-Term Lumen Patency of Vascular Grafts. Bioactive Mater. 6, 1791–1809. 10.1016/j.bioactmat.2020.11.028 PMC7721596 33336112

